# The First Insight into the Tissue Specific *Taxus* Transcriptome via Illumina Second Generation Sequencing

**DOI:** 10.1371/journal.pone.0021220

**Published:** 2011-06-22

**Authors:** Da Cheng Hao, GuangBo Ge, PeiGen Xiao, YanYan Zhang, Ling Yang

**Affiliations:** 1 Biotechnology Institute, Dalian Jiaotong University, Dalian, China; 2 Pharmaceutical Resource Discovery, Dalian Institute of Chemical Physics, Chinese Academy of Sciences, Dalian, China; 3 Institute of Medicinal Plant Development, Chinese Academy of Medical Sciences, Beijing, China; University of Uppsala, Sweden

## Abstract

**Background:**

Illumina second generation sequencing is now an efficient route for generating enormous sequence collections that represent expressed genes and quantitate expression level. *Taxus* is a world-wide endangered gymnosperm genus and forms an important anti-cancer medicinal resource, but the large and complex genomes of *Taxus* have hindered the development of genomic resources. The research of its tissue-specific transcriptome is absent. There is also no study concerning the association between the plant transcriptome and metabolome with respect to the plant tissue type.

**Methodology/Principal Findings:**

We performed the *de novo* assembly of *Taxus mairei* transcriptome using Illumina paired-end sequencing technology. In a single run, we produced 13,737,528 sequencing reads corresponding to 2.03 Gb total nucleotides. These reads were assembled into 36,493 unique sequences. Based on similarity search with known proteins, 23,515 Unigenes were identified to have the Blast hit with a cut-off E-value above 10^−5^. Furthermore, we investigated the transcriptome difference of three *Taxus* tissues using a tag-based digital gene expression system. We obtained a sequencing depth of over 3.15 million tags per sample and identified a large number of genes associated with tissue specific functions and taxane biosynthetic pathway. The expression of the taxane biosynthetic genes is significantly higher in the root than in the leaf and the stem, while high activity of taxane-producing pathway in the root was also revealed via metabolomic analyses. Moreover, many antisense transcripts and novel transcripts were found; clusters with similar differential expression patterns, enriched GO terms and enriched metabolic pathways with regard to the differentially expressed genes were revealed for the first time.

**Conclusions/Significance:**

Our data provides the most comprehensive sequence resource available for *Taxus* study and will help define mechanisms of tissue specific functions and secondary metabolism in non-model plant organisms.

## Introduction

*Taxus* is a genus of yews, small coniferous trees or shrubs in the gymnosperm family Taxaceae. There are at least 14 species in *Taxus* [1], [2], most of which are the sources of biological active compounds such as paclitaxel or Taxol, a chemotherapeutic drug used in the treatment of many types of cancer. The increasing demands have created a supply crisis and raised serious environmental concerns [3]. Presently the mainstay solution is semisynthesis from several precursors which have the same core skeleton and can be isolated from renewable yew resources [4], [5]. Paclitaxel and its precursors belong to a group of typical secondary metabolites named the taxane diterpenoids or taxoids, but the distribution and content are highly varied with species and tissues. For that reason, choosing suitable *Taxus* species and screening the constituents in each tissue are essential to cost-effective production of taxane drugs. Previous studies mainly focused on *Taxus* needles from various species origin which displayed distinct chemical distribution [5], [6]. In recent years, we systematically investigate the root constituents from various *Taxus* species and found that roots have relatively simple chemical profiles and possess high yields of valuable taxanes such as paclitaxel (P), cephalomannine (C), 10-deacetylpaclitaxel (10-DAT) and 7-xylosyltaxanes. Rational exploitation of the taxanes that the root contains is of great help for alleviating the taxane supply crisis. However, the biosynthesis pathway of paclitaxel and other taxanes is not fully elucidated and the underlying molecular mechanism of the metabolic difference between different *Taxus* tissues has not been studied, which hamper improvements in taxane drug production.

During the past few years, the high demand for low-cost sequencing has driven the development of high-throughput second generation sequencing technologies that parallelize the sequencing process, producing thousands or millions of sequences at once [7], [8]. Solexa, now part of Illumina developed a sequencing technology based on reversible dye-terminators [9]. The inexpensive production of large volumes of sequence data via second generation sequencing is the primary advantage over conventional methods [10]. Collins et al. [11] used a combination of mapping and *de novo* assembly tools in the analysis of data from Solexa sequencing of the polyploid plant *Pachycladon enysii* and demonstrated that transcriptome analysis using high-throughput short-read sequencing need not be restricted to the genomes of model organisms. The *de novo* transcriptome sequencing and characterization based on Illumina second generation sequencing technology has been performed successfully for sweet potato [12], *Eucalyptus* tree [13], chickpea [14], and orchid [15]. Illumina produces orders of magnitude more sequence at a fraction of the cost of 454 platform. Despite its obvious potential, Illumina second generation sequencing has not been applied to the gymnosperm research.

Besides transcriptome sequencing, another approach to gene expression analysis, digital gene expression (DGE) profiling, can be performed on the Illumina Genome Analyzer sequencing platform. DGE tag sequencing is an implementation of the LongSAGE (serial analysis of gene expression) protocol on the Illumina sequencing platform that increases utility while reducing both the cost and time required to generate gene expression profiles. The ultra-high-throughput sequencing capability of the Illumina platform allows the cost-effective generation of libraries containing an average of 20 million tags, a 200-fold improvement over classical LongSAGE [16]. Illumina DGE has less sequence composition bias, leading to a better representation of AT-rich tag sequences, and allows a more accurate profiling of a subset of the transcriptome characterized by AT-rich genes expressed at levels below the threshold of detection of LongSAGE [17]. Illumina DGE has been used in a sweet orange red-flesh mutant to study the molecular process regulating lycopene, a secondary metabolite, accumulation [18]. Lee et al. [19] compared the expression profiles of cultured dedifferentiated *Taxus cuspidata* cells and undifferentiated cambial meristematic cells and identified marker genes and transcriptional programs consistent with a stem cell identity. However, the expression pattern of the genes involved in the taxane biosynthesis was not reported. The reference transcriptome for the DGE tag mapping is obtained with 454 pyrosequencing and thus is relatively small (301 Mb of sequence).

The main goal of this study is, first, to create a backbone for the *Taxus* and gymnosperm research community, including large part of the transcriptome as well as expression patterns in different tissues; second, to look at phenotype and try to look at correlation between gene expression and taxane metabolites. In this study, we generated over two billion bases of high-quality DNA sequence with Illumina technology and demonstrated the suitability of short-read sequencing for de novo assembly and annotation of genes expressed in a gymnosperm with little prior genome information [20]. In a single run, we identified 36,493 distinct Unigenes including hundreds of taxane biosynthetic genes. Furthermore, we compared the gene expression profiles of three taxane-producing *Taxus* tissues using a digital gene expression (DGE) system. The assembled, annotated transcriptome sequences and gene expression profiles provide an invaluable resource for the identification of *Taxus* genes involved in taxane biosynthesis and tissue specific functions.

## Results

### Transcriptome sequencing (mRNA-seq) output, assembly, expression annotation

**Figure 1:**
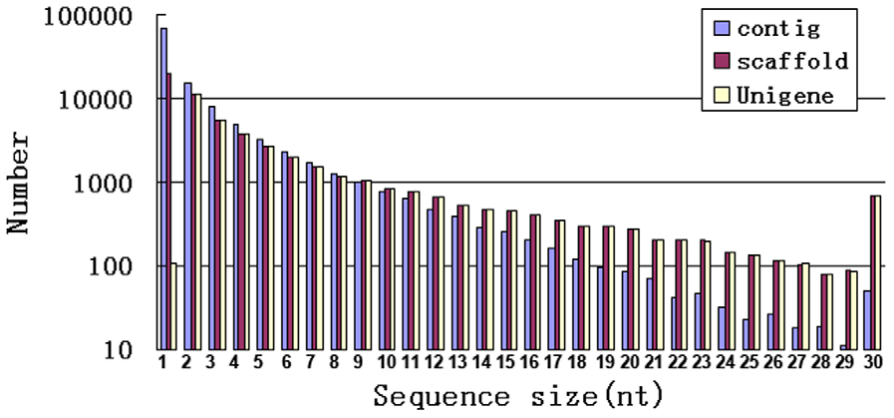
Statistics of Illumina short read assembly quality. The length distribution of the *de novo* assembly for contigs, scaffolds and Unigenes is shown. 1, 200; 2, 300; 3, 400; 4, 500; 5, 600; 6, 700; 7, 800; 8, 900; 9, 1,000; 10, 1,100; 11, 1,200; 12, 1,300; 13, 1,400; 14, 1,500; 15, 1,600; 16, 1,700; 17, 1,800; 18, 1,900; 19, 2,000; 20, 2,100; 21, 2,200; 22, 2,300; 23, 2,400; 24, 2,500; 25, 2,600; 26, 2,700; 27, 2,800; 28, 2,900; 29, 3,000; 30, >3,000.

By Illumina high-throughput second generation sequencing, 13,737,528 reads were obtained, with total nucleotides 2,033,154,144 (2.03 Gb). The average read size, Q20 percentage (sequencing error rate <1%), and GC percentage are 148 bp, 96.48%, and 45.54%, respectively. From these short reads 112,769 contigs were assembled, with median length of 384 bp (Fig. 1). From contigs 56,393 scaffolds were made by SOAPdenovo, with median length of 928 bp (Fig. 1). From scaffolds 36,493 Unigenes were obtained with median length of 1,077 bp (Fig. 1, Table S1). There is no complete gymnosperm genome available, therefore we use the four sequence datasets, i.e., 201,405 clone sequences and 27,720 cluster sequences (Unigenes) of *Picea glauca* from http://www.arborea.ulaval.ca/research/sequencing/gene_catalogue/index.html, 36,906 Unigene sequences of a Korea *Taxus cuspidata* [19], and 20,557 Unigene sequences (12,975 singletons and 7,582 contigs) of a China *T. cuspidata* [21], to add annotation against gymnosperm species (Table S2). With the alignment parameters E value ≤1e-5 and the sequence identity ≥80%, 44.6%, 43.8%, 75.3%, and 49.8% of *T. mairei* Unigenes were annotated with clone sequences and Unigenes of *P. glauca*, Unigenes of Korea *T. cuspidata*, and Unigenes of China *T. cuspidata*, respectively. The RPKM method is able to eliminate the influence of different gene length and sequencing level on the calculation of gene expression [22]. The median RPKM value of all Unigenes is 15.73, while the minimal and maximal values are 0 (nine Unigenes) and 5,464.39 (Unigene 15,622), respectively. Twenty six Unigenes has RPKM values of more than 1,000 (Table S1), many of which may be involved in plant defense. Seventy six Unigenes has RPKM values of less than 0.4, implying the potential of mRNA-seq in detecting genes of extremely low expression.

### Functional annotation

Distinct gene sequences were first searched using BLASTx against the NCBI non-redundant (nr) database using a cut-off E-value of 10^−5^. Using this approach, 23,515 Unigenes (64.4% of all Unigenes) returned an above cut-off BLAST result (Table S1). Due to lack of genome and EST information in *Taxus*, 35.6% of Unigenes could not be matched to known genes. Similarly, up to 19,869 Unigenes (54.4% of all) had no Swissprot annotation.

**Figure 2:**
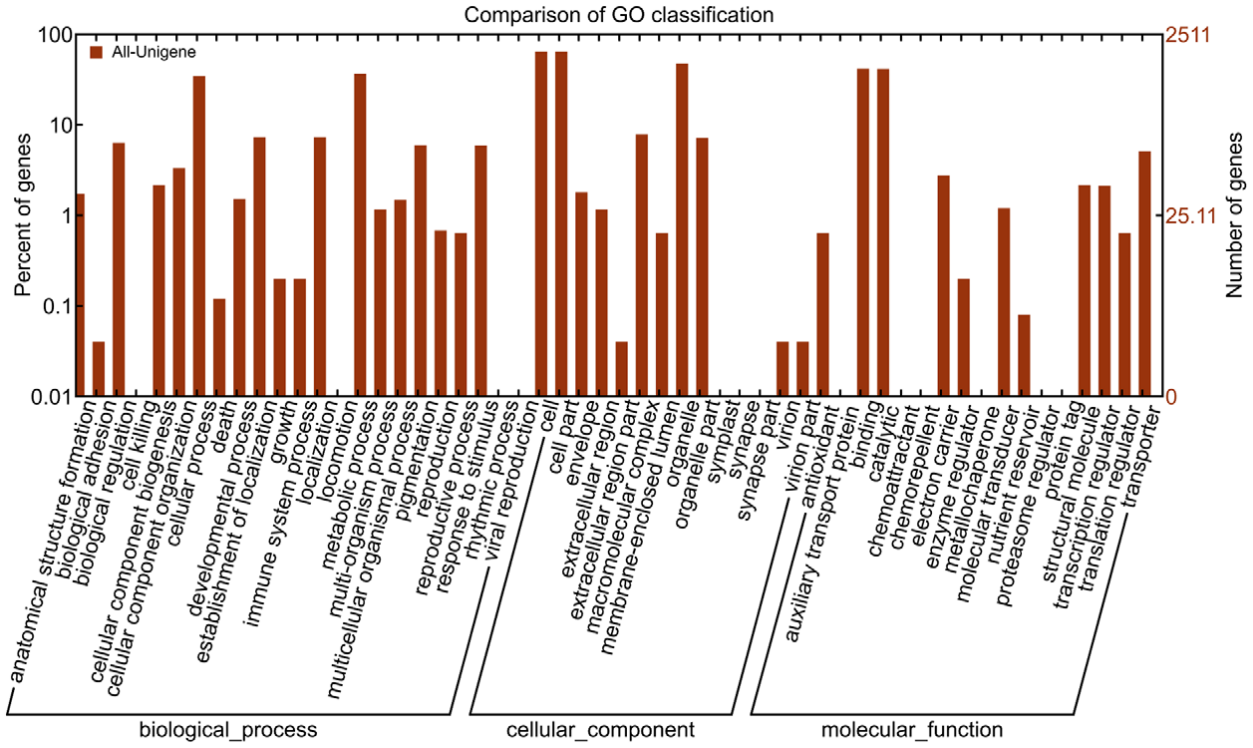
Histogram presentation of Gene Ontology classification. The results are summarized in three main categories: biological process, cellular component and molecular function. The right y-axis indicates the number of genes in a category. The left y-axis indicates the percentage of a specific category of genes in that main category.

**Figure 3:**
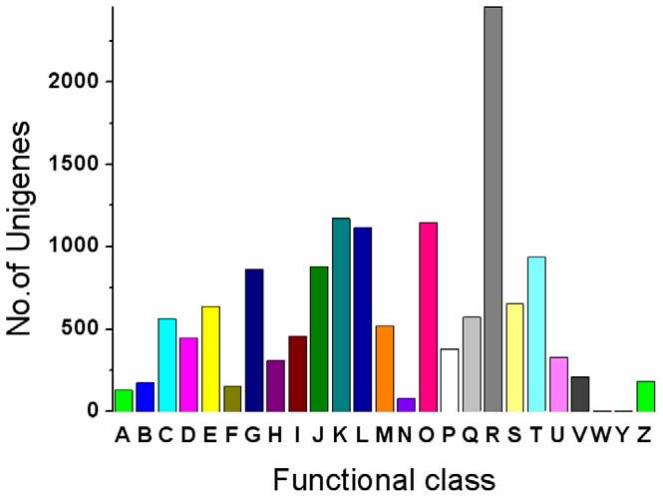
Histogram presentation of clusters of orthologous groups (COG) classification. Out of 23515 nr hits, 14,381 sequences have a COG classification among the 25 categories. A, RNA processing and modification; B, Chromatin structure and dynamics; C, Energy production and conversion; D, Cell cycle control, cell division, chromosome partitioning; E, Amino acid transport and metabolism; F, Nucleotide transport and metabolism; G, Carbohydrate transport and metabolism; H, Coenzyme transport and metabolism; I, Lipid transport and metabolism; J, Translation, ribosomal structure and biogenesis; K, Transcription; L, Replication, recombination and repair; M, Cell wall/membrane/envelope biogenesis; N, Cell motility; O, Posttranslational modification, protein turnover, chaperones; P, Inorganic ion transport and metabolism; Q, Secondary metabolites biosynthesis, transport and catabolism; R, General function prediction only; S, Function unknown; T, Signal transduction mechanisms; U, Intracellular trafficking, secretion, and vesicular transport; V, Defense mechanisms; W, Extracellular structures; Y, Nuclear structure; Z, Cytoskeleton.

Gene ontology (GO) assignments were used to classify the functions of the predicted *Taxus mairei* genes. Based on sequence homology, 10,263 sequences can be categorized into 54 functional groups (Fig. 2). In each of the three main categories (biological process, cellular component and molecular function) of the GO classification, “metabolic process”, “cell part” and “binding” terms are dominant respectively. We also noticed a high-percentage of genes from categories of “cellular process”, “organelle” and “catalytic” and only a few genes from terms of “nutrient reservoir”, “extracellular region part” and “death” (Fig. 2). To further evaluate the completeness of our transcriptome library and the effectiveness of our annotation process, we searched the annotated sequences for the genes involved in COG classifications. In total, out of 23,515 nr hits, 14,381 sequences have a COG classification (Fig. 3). Among the 25 COG categories, the cluster for “general function prediction” represents the largest group (2,458, 17.1%) followed by “transcription” (1,173, 8.2%) and “posttranslational modification, protein turnover, chaperones” (1,146, 8.0%). The following categories: extracellular structures (2, 0.014%); nuclear structure (3, 0.02%) and cell motility (78, 0.5%), represent the smallest groups (Fig. 3). To identify the biological pathways that are active in *Taxus mairei*, we mapped the 23,515 annotated sequences to the reference canonical pathways in Kyoto Encyclopedia of Genes and Genomes (KEGG) [23]. In total, we assigned 11,550 sequences to 124 KEGG pathways. The pathways with most representation by the unique sequences were “metabolic pathways” (2,867 members); “biosynthesis of plant hormones” (730 members) and “spliceosome” (675 members). In secondary metabolism subclass, most transcripts were mapped to “biosynthesis of phenylpropanoids” (611), “biosynthesis of alkaloids derived from shikimate pathway” (309), “flavonoid biosynthesis” (254), “carotenoid biosynthesis” (156), “diterpenoid biosynthesis” (94), “metabolism of xenobiotics by cytochrome P450” (90), and “terpenoid backbone biosynthesis” (69). These annotations provide a valuable resource for investigating specific processes, functions and pathways and allow for the identification of novel genes involved in the pathways of secondary metabolite synthesis.

### Detection of sequences related to the taxane biosynthetic pathway and metabolism

In plants, the plastid-localized 2-C-methyl-d-erythritol 4-phosphate (MEP) pathway provides the precursors for the synthesis of terpenes. All genes encoding the seven enzymes in the plastidial MEP pathway [24] were identified in the Illumina datasets (Table S3). The sequences encoding 1-deoxy-D-xylulose-5-phosphate synthase (DXS), the first enzyme of the MEP pathway, were substantially abundant with RPKM 910.6. The two enzymes catalyzing the slow steps of the mevalonate-independent pathway, 4-hydroxy-3-methylbut-2-en-1-yl diphosphate synthase (HDS) and 4-hydroxy-3-methylbut-2-enyl diphosphate reductase (HDR), held RPKM 496.3 and 365.7, respectively. The downstream IPP isomerase (IDI) and geranylgeranyl diphosphate synthase (GGPPS) held RPKM 173.2 and 787.7, respectively. The reaction catalyzed by GGPPS transforms the IPP and the isomer DMAPP into the GGPP, which plays a pivotal role in the isoprenoid metabolism and the taxane biosynthetic pathway. The downstream enzyme of DXS, DXP reductoisomerase (DXR; RPKM 168.3), and GGPPS were quite upregulated in methyl jasmonate-induced *Taxus* cells [25]. For the first time, all 13 known genes involved in the paclitaxel biosynthesis were identified in the Illumina datasets. As an enzyme of the early step, taxane 10β-hydroxylase (T10OH), with RPKM 2611.2, was well represented (Table S3). Taxadiene synthase (TS), with RPKM 2,106.5, catalyzes the cyclization of the linear isoprenoid substrate GGPP to form taxa-4(5),11(12)diene. Taxane 2α-O-benzoyltransferase (TBT), involved in the production of 10-deacetylbaccatin III (10-DAB) in the middle step, held RPKM 473.0. Taxoid 14β-hydroxylase (T14OH), which is responsible for diversion of the pathway to 14-hydroxy taxoids that are prominent metabolites of *Taxus* cell cultures [26], held RPKM 623.2 in the present study. Although the biosynthesis of paclitaxel and related taxoids are beginning to be understood, many steps still remain to be addressed. The C1β-hydroxylation and the C4,C20β-epoxidation leading to oxetane ring formation, oxidation of the C9-hydroxyl group to the corresponding carbonyl in the taxane core formation, CoA-dependent ligation to β-phenylalanine, 2′-hydroxylation of N-debenzoyl-2′-deoxytaxol, and N-benzoylation of N-debenzoyltaxol in the side-chain assembly are the remaining steps [25], [27]. Available evidence suggests that C4, C20β-epoxidation occurs at the level of a taxadien-hexaol and is followed by ring expansion toward oxetane construction [28]. These steps could be mediated by cytochrome P450 (CYP). In the side-chain assembly, the catalyzed transformation of 2′-hydroxylation of β-phenylalanoyl baccatin III (N-debenzoyl-2′-deoxytaxol) into phenylisoserinoyl baccatin III (N-debenzoyltaxol) implies the involvement of CYP [29]. Meanwhile, paclitaxel biosynthesis competes with side routes produced by numerous acyl/aroyl transferases or the oxidation of the taxane nucleus derived from common precursors. Identification of these side-route genes could have an important implication in eventually increasing paclitaxel yields [30]. For this purpose, we screened our Illumina datasets and discovered 211 candidate genes of CYP, seven epoxidases, 34 CoA ligases, 32 N-benzoyltransferases, 102 glycosyltransferases (GTs), and six xylosyltransferases (XYLTs) (Table S1). These sequences provide useful information for elucidating the remaining steps and exploring the side-route enzymes in paclitaxel biosynthetic pathway.

### Digital gene expression (DGE) library sequencing

**Figure 4:**
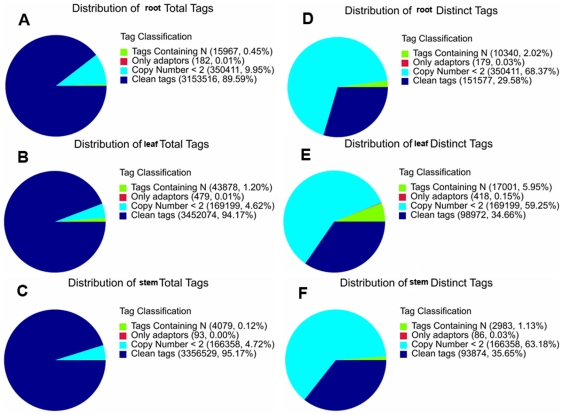
Evaluation of DGE library sequencing quality. A, distribution of root total tags; B, distribution of leaf total tags; C, distribution of stem total tags; D, distribution of root distinct tags; E, distribution of leaf distinct tags; F, distribution of stem distinct tags.

Three DGE libraries were made from the *Taxus* root, stem, and leaf, respectively, and sequenced using Illumina technology. We first evaluate the sequencing quality. The total tags of root, stem, and leaf are 3,520,076, 3,527,059, and 3,665,630, respectively (Table 1, Fig. 4). Clean tags are those used in the analysis after filtering the dirty tags. 94.17% and 95.17% of the total tags in the leaf and the stem are clean tags, respectively, which is higher than that in the root (89.59%) (Fig. 4A–C). More tags with copy number <2 are in the root than in the leaf and the stem (9.95% vs. 4.62%, 4.72%). The leaf library has more tags containing N than the root and the stem libraries (1.20% vs. 0.45%, 0.12%). The distinct tags of root, stem, and leaf are 512,507, 263,301, and 285,590, respectively (Fig. 4D–F). The distribution of the distinct tags in the three tissues is significantly different (p<0.0001). 34.66% and 35.65% of the distinct tags in the leaf and the stem are clean tags, respectively, which is higher than that in the root (29.58%). The leaf library has more distinct tags containing N than the root and the stem libraries (5.95% vs. 2.02%, 1.13%). Despite the above differences, results suggest that the sequencing quality is high enough to allow for the following analyses.

**Table 1:** Mapping DGE library sequences to the reference transcriptome database.

		Root	Stem	Leaf
Raw data	Total	3,520,076	3,527,059	3,665,630
	Distinct tag	512,507	263,301	285,590
Clean tag	Total number	3,153,516	3,356,529	3,452,074
	Distinct tag number	151,577	93,874	98,972
All tag mappingto gene	Total number	1,400,670	1,420,764	1,575,977
	Total % of clean tag	44.42%	42.33%	45.65%
	Distinct tag number	50,958	40,579	41,072
	Distinct tag % of clean tag	33.62%	43.23%	41.50%
Unambiguous tagmapping to gene	Total number	1,388,905	1,412,459	1,564,537
	Total % of clean tag	44.04%	42.08%	45.32%
	Distinct tag number	50,568	40,313	40,785
	Distinct tag % of clean tag	33.36%	42.94%	41.21%
All tag-mappedgenes	Number	17,477	16,142	16,314
	% of ref genes	47.89%	44.23%	44.70%
Unambiguous tag-mapped genes	Number	17,285	15,966	16,163
	% of ref genes	47.37%	43.75%	44.29%

**Figure 5:**
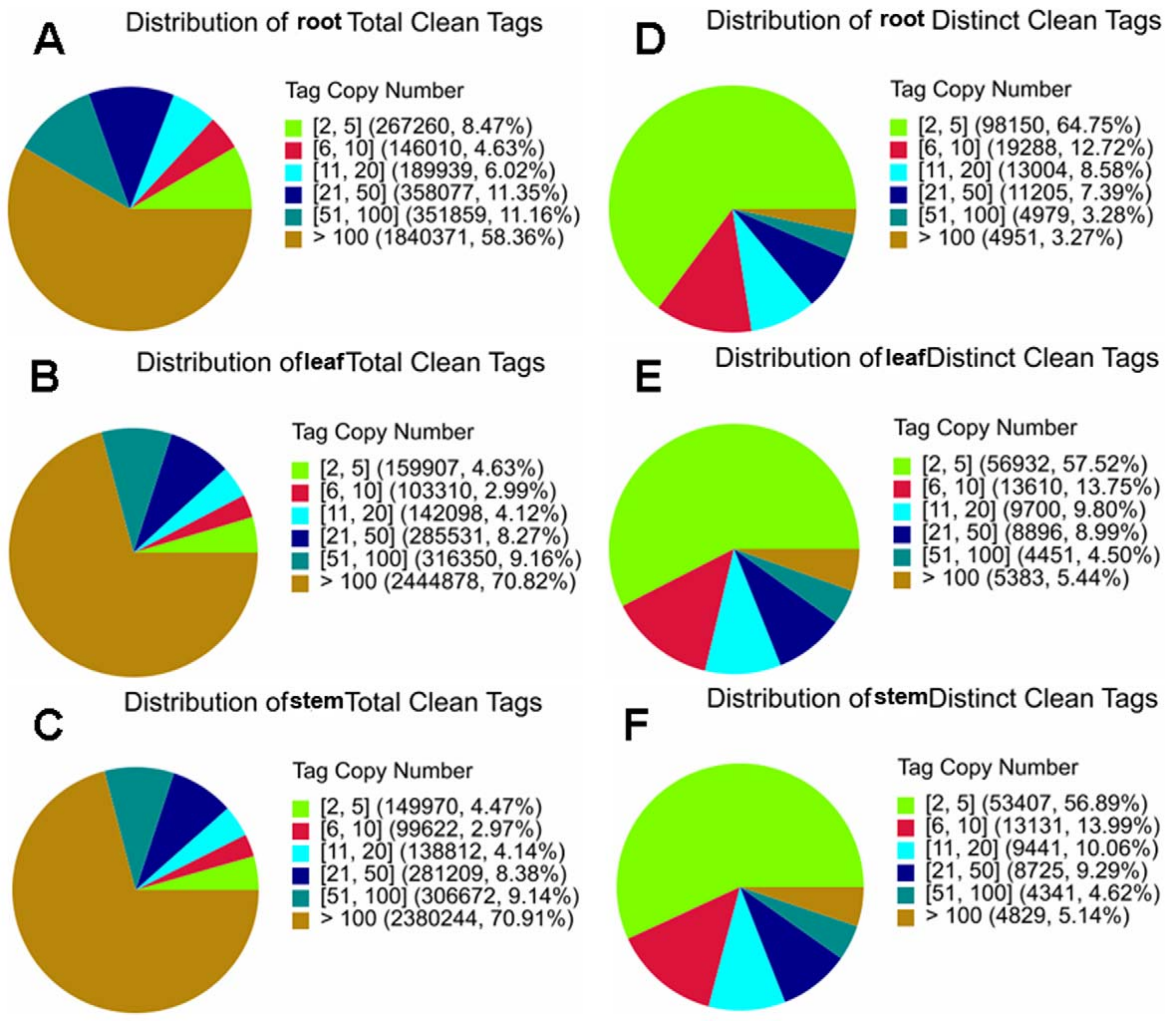
Distribution of total tags and distinct tags over different tag abundance categories. Distribution of the total clean tags in the root (A), leaf (B), and stem (C). Numbers in the square brackets indicate the range of copy numbers for a specific category of tags. For example, [2], [5] means all the tags in this category has 2 to 5 copies. Numbers in the parentheses show the total tag copy number for all the tags in that category. Distribution of the distinct clean tags in the root (D), leaf (E), and stem (F). Numbers in the square brackets indicate the range of copy numbers for a specific category of tags. Numbers in the parentheses show the total types of tags in that category.

The saturation analysis was performed to check whether the number of detected genes keep increasing when sequencing amount (total tag number) increases (Fig. S1). When sequencing amount of three DGE libraries reaches 2 M or higher, the number of detected genes almost ceases to increase. Heterogeneity and redundancy are two significant characteristics of mRNA expression. Small number categories of mRNA have very high abundance, while the rest majority stays at a very low level of expression. The distribution of clean tag expression can be used to evaluate the normality of the whole DGE data (Fig. 5). The clean tags of the root, the stem, and the leaf are 3,153,516, 3,356,529, and 3,452,074, respectively (Table 1, Fig. 5A–C). The distribution of the clean tags in the three libraries is significantly different (p<0.0001). 70.82% and 70.91% of the clean tags in the leaf and the stem possess more than 100 copies, respectively, which is higher than that in the root (58.36%). 8.47% of the clean tags in the root possess copies between two and five, while only 4.63% and 4.47% of the clean tags in the leaf and the stem have such a low abundance, respectively. There are 151,577, 93,874, and 98,972 distinct clean tags in the root, stem, and leaf, respectively (Fig. 4 and Fig. 5D–F). The distribution of the distinct clean tags in the three tissues is significantly different (p<0.0001). 64.75% of the distinct clean tags in the root have low copy numbers between two and five, which is higher than those in the leaf (57.52%) and the stem (56.89%). DGE method generates absolute rather than relative gene expression measurements and avoids many inherent limitations of microarray analysis.

### Mapping sequences to the reference transcriptome database

To reveal the molecular events behind DGE profiles, we mapped the tag sequences of the three DGE libraries to our transcriptome reference database that contains 36,493 Unigene sequences. There are 117,847 unambiguous reference tags, i.e., 99.54% of all reference tags. 81.54% (29,757) of Unigenes possess CATG restriction site of *Nla*III enzyme used in the tag library construction. Among the 93,874 (stem) to 151,577 (root) distinct tags generated from the Illumina sequencing of the three libraries, 40,313 (stem) to 50,568 (root) distinct tags were mapped to a gene in the reference database (Table 1, Fig. S2). Tags mapped to a unique sequence are the most critical subset of the DGE libraries as they can explicitly identify a transcript. Up to 50.7% (18,512) of the sequences in our transcriptome reference database could be unequivocally identified by unique tag (Table S4). Next, the level of gene expression was determined by calculating the number of unambiguous tags for each gene and then normalizing this to the number of transcripts per million tags (TPM) (Fig. S3). The dynamic range of DGE spanned five orders of magnitude, i.e., from zero to 36,696 tag counts. However, the tag counts for the majority of genes were low in the three libraries. The median tag counts are only 18, 14, and 14 in the root, stem, and leaf, respectively (Table S4), suggesting the ability of DGE in detecting rare transcripts. Fig. S3 shows that the mRNA transcribed from the major kinds of genes is represented in fewer than 315 tags (about TPM 100) and only a small proportion of genes are highly expressed. The frequency distribution of gene expression in the leaf is more similar to that in the stem than that in the root.

### Differentially expressed genes (DEGs), antisense transcripts and novel transcripts

**Figure 6:**
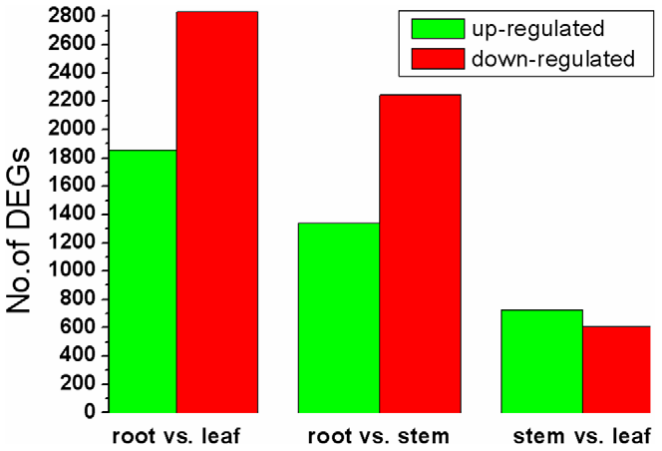
Changes in gene expression profile among the different *Taxus* tissues. The number of up-regulated and down-regulated genes between root and leaf, root and stem, and stem and leaf are summarized. For example, “1,854” means that the expression of 1,854 genes is statistically significantly higher in the leaf than in the root; “2,830” means that the expression of 2,830 genes is statistically significantly lower in the leaf than in the root.

To identify genes showing a significant expression difference in different *Taxus* tissues, the differentially expressed tags between two samples were identified by an algorithm modified from Audic and Claverie [31]. A total of 15,660 significantly changed tag entities were detected between the root and leaf libraries. Those tags were mapped to 6,740 genes with 1,854 genes up-regulated (higher expression in the leaf) and 2,830 genes down-regulated (Fig. 6, Table S5). Between the root and stem libraries, a total of 3,584 DEGs were detected with less up-regulated genes (1,339) and more down-regulated genes (2,245) (Table S6). Between the stem and leaf libraries, 1,332 DEGs were detected with 726 up-regulated genes and 606 down-regulated genes (Table S7). This suggests that there are more DEGs between root and leaf and between root and stem than those between stem and leaf. Next we analyzed the expression of antisense transcripts in the three tissues. Sense-antisense plays an important role on gene expression regulation. Sequencing tags mapped to the complementary strand of the sense gene suggest that its antisense strand also has transcripts, and this gene may use the sense-antisense regulation. The antisense transcripts were detected in 8,954, 8,597, and 9,563 Unigenes of the root, stem, and leaf libraries, respectively (Table 2). 21.3%–22.6% of these genes are orphan sequences - no homologues found in the NCBI database. 25.6%–26.6% of Unigenes with TPM(+)/TPM(−) ≥1 are orphan sequences, which might be consistent with the expectation that *Taxus mairei* expresses genes with no homologues in other species. 3.99%–5.49% of Unigenes with TPM(+)/TPM(-) <1 are orphan sequences, which might be functional in the post-transcriptional gene regulation.

**Table 2:** Antisense regulation of genes in the three *Taxus* tissues.

		Root	Stem	Leaf
No. of Unigenes with antisense(−) transcript		8,954	8,597	9,563
TPM(−)	min	0.63	0.6	0.58
	max	1,908.35	3,867.09	5,143.86
	median	1.59	1.49	1.45
	Percentage of sequenceswithout Blast hit	22.6% (2029)	22.1% (1907)	21.3% (2042)
TPM(+)/TPM(−) ratio	min	0	0	0
	max	1,158.30	990.60	1,241.13
	median	7.23	7.34	6.35
No. of Unigenes with TPM(+)/TPM(−)<1		1,310	1,719	2,230
Percentage of sequences without Blast hit		5.49% (72)	4.36% (75)	3.99% (89)
No. of Unigenes with TPM(+)/TPM(−)≥1		7,644	6,878	7,333
Percentage of sequences without Blast hit		25.6% (1957)	26.6% (1832)	26.6% (1953)

TPM(+), Normalized expression level of sense genes; TPM(−), Normalized expression level of antisense genes.

It is unknown whether the expression profiles of paclitaxel biosynthetic genes differ in the three *Taxus* tissues, therefore we analyzed the DEGs involved in this secondary metabolism pathway. Comparing the root and the leaf, we found the expression of 23 Unigenes was significantly higher in the root than in the leaf (FDR ≤0.001 and ∣log_2_Ratio∣≥1, Tables 3 and S8), while only six Unigenes had significantly lower expression in the root than in the leaf. Similarly, the expression of 29 Unigenes was much higher in the root than in the stem, while only one Unigene had much lower expression in the root than in the stem. Sixteen Unigenes were expressed less abundantly in both the leaf and the stem than in the root, including Unigene 35278 (GGPPS), Unigenes 11020 and 28660 (taxadiene 5α hydroxylase, T5OH), Unigene 32910 (taxadien-5α-ol O-acetyltransferase, TAT), Unigenes 13330, 1373, 8115, and 26700 (T10OH), Unigene 14478 (taxoid 2α hydroxylase, T2OH), Unigene 17688 (taxoid 7β hydroxylase, T7OH), Unigene 2582 (TBT), Unigene 11534 (phenylpropanoyl transferase, BAPT), Unigenes 15942, 24051, and 5777 (3′-N-debenzoyl-2′-deoxytaxol N-benzoyltransferase, DBTNBT), and Unigene 21200 (phenylalanine aminomutase, PAM). In contrast, the expression of 61 Unigenes was comparable between the stem and the leaf, while only eight Unigenes had much lower expression in the stem than in the leaf. These results imply that the expression level of genes involved in the paclitaxel biosynthetic pathway is higher in the root than in the leaf and the stem, which might result in more enzyme products and higher enzyme activities in the root. Since the antisense regulation is a ubiquitous mechanism, we also analyzed whether there is potential antisense regulation involved in the paclitaxel biosynthetic pathway. Forty two, 25, and 37 Unigenes in the root, stem, and leaf, respectively, were found to have antisense expression, ranging from TPM 0.58 to TPM 10.15 (Table S9). Nine, eight, and 14 Unigenes in the root, stem, and leaf, respectively, were found to have sense/antisense expression ratio of not more than one. For example, Unigene 35249, representing the DXS of the MEP pathway, had antisense expression but no sense expression in all three tissues; Unigene 14905, the T10OH of the paclitaxel biosynthetic pathway, had antisense expression but no sense expression in the leaf and the stem. The role of antisense expression in the regulation of gene expression is not well known especially not outside model species. It should be noted that what is detected as antisense transcripts could belong to the transcript belonging to another gene transcribed on the other strand as well as misplaced short reads.

**Table 3:** Number of DEGs involved in the taxane biosynthetic pathway.

	Root vs. leaf				Root vs. stem				Stem vs. leaf			
	TPM root>leaf		TPM root<leaf		TPM root>stem		TPM root<stem		TPM stem>leaf		TPM stem<leaf	
	significant	NS	significant	NS	significant	NS	significant	NS	significant	NS	significant	NS
paclitaxel biosynthetic pathway	18	26	5	17	19	34	1	9	0	15	6	33
MEP pathway	3	11	1	2	8	7	0	2	0	1	2	9
XYLT	2	1	0	0	2	1	0	0	0	2	0	1
Sum	23	38	6	19	29	42	1	11	0	18	8	43

We use “FDR ≤0.001 and the absolute value of log_2_Ratio ≥1” as the threshold to judge the significance of gene expression difference. NS, not statistically significant.

Comparing with microarray, DGE profiling does not need pre-designed probes, and can help detecting new transcripts [32]. Since the whole genome sequences of the *Taxus* is not available, we map the clean tags that cannot be mapped to mRNA to the 1,923 fosmid end sequences (FESs) of *Taxus mairei* genomic library that we constructed [20], by which 1,106, 702, and 748 novel transcripts were predicted in the root, stem, and leaf, respectively (Table S10). The percentages of mismatch and double hits are significantly higher in the *Vitis* genome mapping than in the FES mapping (chi square test, p<0.0001). There are more low abundance transcripts with TPM <5.623 predicted by the FES mapping than by the *Vitis* genome mapping (p<0.0001), while the novel transcripts evenly distribute in the sense and antisense strands despite the mapping methods. Together, 9,678, 5,905, and 5,964 novel transcripts were predicted by the two approaches, providing an invaluable resource for further studies on their functions, protein products, and comparative genomics.

### Clustering analysis of differential gene expression pattern, gene ontology (GO) functional enrichment and pathway enrichment analyses

**Figure 7:**
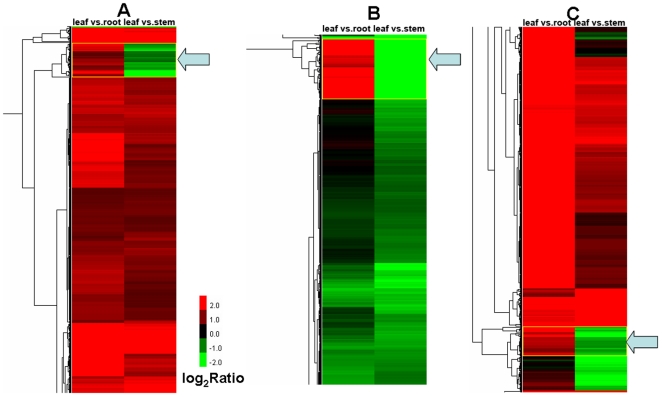
Clusters containing the taxane biosynthetic genes detected in the clustering analyses of DEGs. Each column represents a pair of tissues used in comparison, while each row represents a gene. Expression differences are shown in different colors. Red means up-regulated and green means down-regulated. A, a cluster of 29 Unigenes found in the clustering analysis of the intersection of DEGs (indicated by an arrowhead); B, a cluster of 51 Unigenes found in the analysis of the union of DEGs (indicated by an arrowhead); C, a cluster of 25 Unigenes found in the analysis of the union of DEGs (indicated by an arrowhead).

Genes with similar expression patterns usually mean the functional correlation. In order to screen novel candidate genes that functionally correlate with the taxane biosynthetic pathway, we perform the cluster analysis of gene expression patterns with Cluster [33] software and Java Treeview [34] software. Interestingly, one cluster containing the genes of the taxane biosynthetic pathway was found in the clustering analysis of the intersection of DEGs (Fig. 7A and Table S11). The expression of these genes is coregulated and the expression in the leaf is significantly lower than that in the root but is much higher than that in the stem. This cluster consists of 29 metabolic genes such as Unigenes 26700 and 20390 (T10OH), Unigene 35278 (GGPPS), Unigene 34406 (NADPH-CYP reductase), Unigene 18276 (similar to CYP750A1 of *Pinus*), and Unigene 12035 (similar to CYP720B2 of *Pinus*). Moreover, two clusters containing the genes of the taxane biosynthetic pathway was found in the clustering analysis of the union of DEGs (Fig. 7B, C and Table S11). In one cluster consisted of 51 genes, the expression level of these genes in the leaf is significantly lower than that in the root but is higher than that in the stem. This cluster includes the paclitaxel biosynthetic genes TS (Unigene 13623), BAPT (Unigene 11534), and T10OH (Unigene 8115), as well as other metabolic enzyme genes such as Unigene 8398 (similar to abietadiene synthase of *Abies*), Unigene 32137 (similar to xyloglucan endotransglycosylase), and Unigene 4063 (similar to UDP-glucosyl transferase 85A2). In another cluster of 25 genes, besides six genes (20390, 12035, 35278, 34406, 7568, and 18276) detected in the clustering analysis of the intersection of DEGs, Unigenes 14890 (T10OH), 5510 (TBT), and 23341 (similar to CYP720B1 of *Pinus*) were also found. The expression of these genes in the leaf is significantly lower than that in the root. It is clear that genes of unrelated sequence but similar function cluster tightly together. The genes in the respective cluster are coregulated and are transcribed more actively in a particular tissue, i.e., root. It is probable that many of genes with unknown function and/or unknown protein product will also share common functions with known genes of the same cluster.

**Figure 8:**
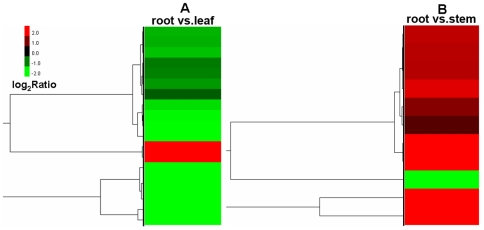
Gene ontology functional enrichment analysis for DEGs. A, the statistically significant term from the molecular function ontology is structural molecule activity in the comparison of the root and the leaf; the cluster image of expression patterns of DEGs under this term is shown. B, the statistically significant term from the cellular component ontology is photosynthetic membrane in the comparison of the root and the stem; the cluster image of expression patterns of DEGs under this term is shown. Each row represents a gene. Expression differences are shown in different colors. Red means up-regulated and green means down-regulated.

The association between GO terms and the lists of DEGs was tested using the GO functional enrichment analysis. The hypergeometric test was used to map all DEGs to terms in the GO database, looking for significantly enriched GO terms in DEGs comparing to the genome (transcriptome) background. In GO molecular function, structural molecule activity is the only significantly enriched GO term in DEGs between the root and the leaf (p = 0.028). The expression of 17 out of 19 genes in this group is significantly higher in the root than in the leaf (Fig. 8A). These genes encode proteins that are structural constituents of the ribosome and the cell wall. There is no significantly enriched GO term in DEGs between the root and the stem and between the leaf and the stem. In GO cellular component, photosynthetic membrane is the only significantly enriched GO term in DEGs between the root and the stem (p = 0.036). Ten out of 11 genes in this group express more abundantly in the stem than in the root (Fig. 8B). There is no significantly enriched GO term in DEGs of other comparisons. This is due, in part, to the fact that functional ontologies used to classify genes are primarily based on bacterial and animal models, and many plant-specific genes have not been functionally annotated. Furthermore, pathway enrichment analysis was performed to further understand the biological functions of DEGs. We mapped all DEGs to terms in the KEGG database and compared this with the whole transcriptome background, with a view to search for genes involved in metabolic or signal transduction pathways that were significantly enriched. Notably, specific enrichment of genes was observed for 13 pathways in the comparison of the root and the leaf (Table S12), with ribosome, biosynthesis of secondary metabolites, and photosynthesis as the top three pathways. Other enriched pathways include phenylpropanoid biosynthesis, phenylalanine metabolism, and flavonoid biosynthesis, et al. Specific enrichment of genes was observed for 16 pathways in the comparison of the root and the stem, with biosynthesis of secondary metabolites, ribosome, and photosynthesis as the top three pathways. The majority of the DEGs were significantly upregulated in the root. In contrast, no specific enrichment of genes was observed for any pathway in the comparison of the leaf and the stem. These results agree the findings from the DEG analysis of the taxane biosynthetic pathway and the clustering analysis and suggest that the secondary metabolism pathway might be more active in the root to provide a larger repertoire of secondary metabolites that are involved in the physiological defense mechanism.

### Phenotypic verification of high activity of taxane-producing pathway in the root via metabolomic analyses

It is postulated that the higher level expression of the taxane biosynthetic genes results in the production of more taxane metabolites in the root. To test this hypothesis, we compare the contents of six representative taxanes synthesized in the roots and the needles with ultra-fast liquid chromatography (UFLC) and mass spectrometry (MS). 10-DAB, baccatin III (B), 10-deacetyl-7-xylosylpaclitaxel (DAXT), 10-DAT, cephalomannine (C), and paclitaxel (P) were detected and quantitated in all root samples and the corresponding needle samples (Tables 4 and 5). The inter-individual variation of the taxane contents is more significant in the needles than in the roots. The most significant variation is in the 10-DAB concentration of the needles, which varied between eight and 301 µg/g dry needle. Despite the inter-individual variation, it is obvious that generally the taxane contents are much higher in the roots than in the needles. The inter-area average content of five out of six representative taxanes is much higher in the roots than in the needles. Since the age of tree influences the taxane content, we further compared the contents of six representative taxanes in the *Taxus mairei* with different ages. All samples were from the same cultivation area in Lishui, Zhejiang province of China. In four out of five age groups, the total taxane amount is much higher in the roots than in the needles (Tables S13 and S14). The inter-group average content of five out of six representative taxanes is much higher in the roots than in the needles, thus enhancing the impression that the *Taxus* roots possess higher activity of taxane-producing pathway.

**Table 4:** Quantitative differences in average concentration of six valuable taxanes in the needles of *Taxus mairei* (from different area).

Collection site(no. of samples)	Taxane content (µg/g dry needle)						
	DAB	B	DAXT	DAT	C	P	Total
Zhejiang (40)	218	53.7	515.6	181.5	89.9	99.6	1,158.3
Fujian (2)	78.9	30.2	314.7	122.1	73.5	73.7	693.1
Jiangxi (1)	222.2	66.4	497.2	210.4	75.2	119.3	1,190.7
Hubei (10)	80.9	152.5	545.1	136.5	91.9	115.3	1,122.2
Average	150.0	75.7	468.2	162.6	82.6	102.0	1,041.1
Range	8–301	21–289	64–1,089	40–269	14–171	42–265	

DAB, 10-deacetylbaccatin III; B, baccatin III; DAXT, 7-xylosyl-10-deacetylpaclitaxel; DAT, 10-deacetylpaclitaxel; C, cephalomannine; P, paclitaxel.

**Table 5:** Quantitative differences in average concentration of six valuable taxanes in the roots of *Taxus mairei* (from different area).

Collection site(no. of samples)	Taxane content (µg/g dry powder)						
	DAB	B	DAXT	DAT	C	P	Total
Zhejiang (22)	149.7	287.2	570.7	351.8	293.4	430.7	2,083.6
Jiangxi (1)	70.2	125.9	399.3	164.5	397.1	470.4	1,627.4
Hubei (10)	153.3	172.8	1113.7	287.0	462.7	574.6	2,764.2
Average	124.4	195.3	694.6	267.76	384.4	491.9	2,158.4
Range	61–238	66–354	531–1,324	145–465	114–750	294–1,012	

DAB, 10-deacetylbaccatin III; B, baccatin III; DAXT, 7-xylosyl-10-deacetylpaclitaxel; DAT, 10-deacetylpaclitaxel; C, cephalomannine; P, paclitaxel.

## Discussion

We have assembled 36,493 expressed gene sequences from an actively growing cultivated *Taxus* tree using Illumina paired-end sequencing (mRNA-seq) technology and *de novo* short read assembly. Quality control comparisons to the Sanger-derived transcript sequences from *Taxus*, as well as multiple lines of evidence such as protein coding sequence (CDS) prediction (Fig. S4) and DGE tag mapping showed that the transcript assemblies are robust and that thousands of coding sequences and their respective 5′ and/or 3′ untranslated regions were successfully assembled (Table S2). For example, 23,145 and 1,860 CDSs were predicted by Blastx and ESTScan, respectively. Among 23,145 predicted CDSs, 14,793 (63.9%) and 14,442 (62.4%) can be aligned with clone sequences and Unigene sequences of *P. glauca*, respectively (data available upon request). Comparison of assembled gene models to gene catalogs of other plant species by Blast analysis and functional annotation (e.g., GO, Swissprot and KEGG) indicate that we have sampled an extensive and diverse expressed gene catalog representing a large proportion of the genes expressed in *Taxus*. Comparison to the few publicly available *Taxus mairei* DNA sequence suggests that we have sampled the most comprehensive set of genes, which is also more complete in length and diversity from a single *Taxus* species than has been available for *Taxus cuspidata* of the genus [19], [21]. Additionally, using DGE to quantify mRNA-seq data we have produced an informative database of transcript abundance across three *Taxus* tissues, which, due to the depth of sequencing, results in much higher sensitivity and wider dynamic range than Sanger or 454-derived EST counts usually associated with this type of analysis.

A concern associated with *de novo* assembly of transcript sequences is the contiguity of assembled sequences. This concern naturally increases as the read length decreases, and may be one of the reasons why most transcriptome *de novo* assembly approaches have utilized technologies with longer read lengths to date. We provide evidence that jointly support the contiguity of transcript sequences assembled in our study using Illumina short-read data. First, a high proportion of the Unigenes exhibited high confidence Blastx similarity to protein sequences from annotated gene catalogs of plant species such as *Arabidopsis*, *Populus* and *Vitis* (Table S1), although one may argue that good hits with Blastx is not any conclusive evidence for correct assemblies. Second, a large proportion of the Unigenes contained long predicted CDSs (Fig S4, Table S2). For example, 4,131 out of 25,005 CDSs (16.5%) predicted by Blastx and ESTScan possess no less than 1,000 bp. The assembly quality and annotation of these sequences could be improved in future by even deeper sequencing and the addition of data from new tissue types. *De novo* assembled transcriptome datasets lack the ability to discriminate and classify the lower confidence annotations, a challenge that is beyond the scope of this study.

The results of clustering analysis of differential gene expression pattern, GO functional enrichment analysis, and KEGG pathway enrichment analysis lend support to the biological significance of DGE profiles derived from short-read sequencing technology, which will assist in the discovery and annotation of novel *Taxus* genes playing key roles in growth and physiology, and particularly in taxane production. The *Taxus* genomic resource produced from this study, as well as future comparative analysis with other gymnosperm species such as *Pinus* and *Picea*, will be valuable for studying the unique biology of this evolutionarily ancient lineage.

**Figure 9:**
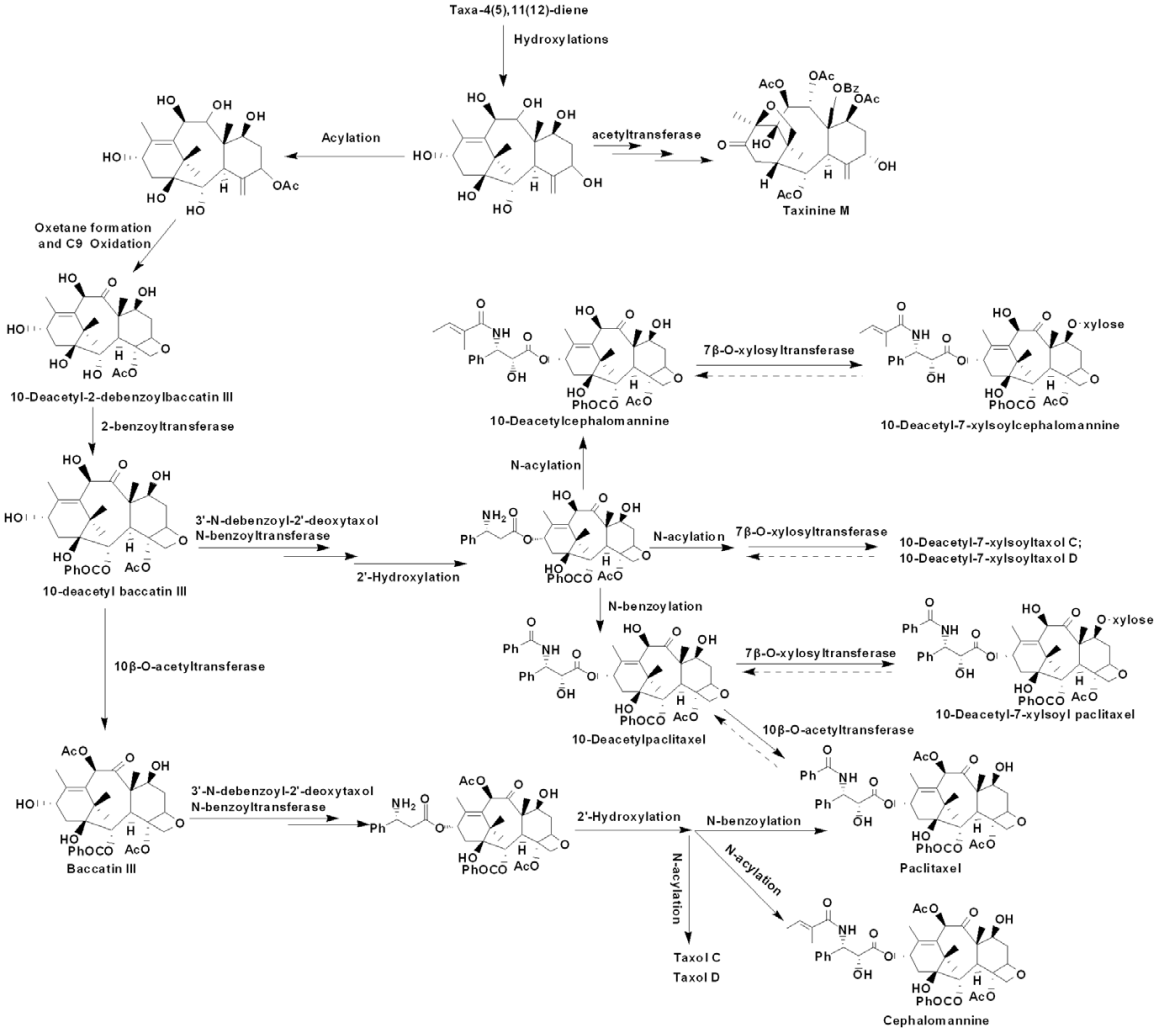
Proposed metabolic framework for taxane biosynthesis in the *Taxus* root. The committed step of Taxol (paclitaxel) biosynthesis is catalyzed by taxa-4(5),11(12)-diene synthase (TS), a diterpene cyclase responsible for transforming the ubiquitous isoprenoid intermediate geranylgeranyl diphosphate (GGPP) to the parent olefin with a taxane skeleton. Taxadiene is then functionalized by a series of eight CYP-mediated oxygenations, three CoA-dependent acylations, and several other transformations *en route* to baccatin III, to which the side chain at C13 is appended to afford final product Taxol. Besides Taxol, *Taxus* species produce about 400 different taxoids, all of which are based upon the unique taxane skeleton. Xylosyltransferases (XYLTs) catalyze the addition of xylosyl group to 10-deacetylpaclitaxel (10-DAT), 10-deacetylcephalomannine (10-DAC), and other 10-deacetyl taxoids transformed from 10-deacetyl baccatin III (10-DAB). These taxoid metabolites may represent defense compounds, and they also constitute significant side-routes that divert pathway flux away from Taxol to decrease production yields of the target drug.

It is found that root samples from different *Taxus* species have similar profiles and nearly identical chemical distributions [4], which suggest that *Taxus* roots have similar metabolic framework. The most significant chemical characteristic of this tissue is its abundant distribution of 7-xylosyltaxanes (Fig. 9), demonstrating that the biosynthesis of 7-xylosyltaxanes is a major metabolic pathway in this plant part. Besides 7-xylosyltaxanes, many essential and valuable taxanes, e.g., 10-DAB, baccatin III, 10-DAT, 10-deacetylcephalomannine (10-DAC), cephalomannine and paclitaxel, are also present in relatively high levels, demonstrating the high competence of taxane synthesis in the *Taxus* root. Studies at the molecular and metabolic levels are thus complementary and cross-validated. It is noted that 33 taxoids [4] with the 10-DAB skeleton exist in the *Taxus* root, which can be used as the semi-synthetic precursors for paclitaxel. These taxoids are also recognized as intermediates or products involved in side routes of paclitaxel formation, i.e. downstream metabolites via the important intermediate 10-DAB during taxane biosynthesis [27]. Furthermore, these pharmaceutically important taxanes comprise over 55% of the peak areas in the entire chromatogram (0–19 min). This is quite different from the needles that usually have major divergent pathways apart from paclitaxel biosynthesis such as the formation of abundant taxine B and taxinine M [35]. The presence and connectivity of all spotted taxanes in the *Taxus* root can be used to figure out the downstream biosynthetic framework of paclitaxel and its analogues such as 10-DAT, cephalomannine, and Taxol C, as well as 7-xylosyltaxanes (Fig. 9). In addition, these detected taxanes and their connectivity facilitate further studies on cell culture and metabolomic analysis of the *Taxus* root.

From an application perspective, the consistent chemical profile suggests that the *Taxus* root resource can be processed by a universal developmental proposal without considering species origin. Moreover, contrary to the needles, the *Taxus* roots have relatively simple chemical constituents and can supply large quantities of various valuable taxanes such as paclitaxel, cephalomannine and 7-xylosyltaxanes. The concentration of paclitaxel and cephalomannine in the roots is two to eight times higher than in the corresponding needles (Tables 4 and 5). Hence the development prospect of the *Taxus* root is very favorable.

In conclusion, this sequence collection represents the first major genomic resource for *Taxus mairei*, and the large number of genes in the different *Taxus* tissues characterized by DGE technology should contribute to further research in this and other gymnosperm species. The *de novo* transcriptome and DGE analyses also provided us with a genome-wide view of the transcriptional and post-transcriptional mechanisms generating an increased number of transcript isoforms in the respective *Taxus* tissue. The data consistency from multiple approaches including transcriptome and metabolome assures that the mRNA-seq and DGE data produced in this study are reliable. Our results illustrate the utility of Illumina second generation sequencing as a basis for defining the metabolic pathway and tissue specific functional genomics in non-model plant species.

## Materials and Methods

### Tissue collection and RNA isolation

Leaf, stem, and root were collected from a cultivated *Taxus mairei* of two years old. Total RNA was isolated with TRIzol (Invitrogen) from each sample according to the manufacturer's instructions. It was treated with RNase-free DNase I (New England BioLabs) for 30 min at 37°C to remove residual DNA.

### cDNA library preparation and sequencing (mRNA-seq)

Beads with oligo(dT) were used to isolate poly(A) mRNA after total RNA was collected from *Taxus* tissues. Fragmentation buffer was added for interrupting mRNA to short fragments. Taking these short fragments as templates, random hexamer-primer was used to synthesize the first-strand cDNA. The second-strand cDNA was synthesized using buffer, dNTPs, RNase H and DNA polymerase I, respectively. Paired-end library was synthesized using the Genomic Sample Prep kit (Illumina, San Diego, CA) according to manufacturer's instructions. Short fragments were purified with QiaQuick PCR extraction kit and resolved with EB buffer for end repair and adding poly(A). After that, the short fragments were connected with sequencing adapters. For amplification with PCR, we selected suitable fragments as templates, with respect to the result of agarose gel electrophoresis. At last, the library was sequenced using Illumina GA IIX. The transcriptome datasets are available at the NCBI Sequence Read Archive (SRA) with the accession number SRX037160.

### Analysis of Illumina transcriptome sequencing results

The pipeline of the transcriptome bioinformatic analysis is shown in Fig. S5. Sequencing-received raw image data is transformed by base calling into sequence data, which is called raw data or raw reads, and is stored in fastq format. This type of files stores reads sequences and quality. Raw reads that only have 3′ adaptor fragments were removed before data analysis. All following analysis are based on clean reads. Transcriptome *de novo* assembly is carried out with short reads assembling program – SOAPdenovo [36]. SOAPdenovo firstly combines reads with certain length of overlap to form longer fragments without N, which are called contigs. While the raw read length from the Illumina GAIIx is 76 bp, SOAPdenovo provides the assembly results with contigs (and Unigenes) of no shorter than 200 bp. Then the reads are mapped back to contigs; with paired-end reads it is able to detect contigs from the same transcript as well as the distances between these contigs. Next, SOAPdenovo connects the contigs using N to represent unknown sequences between each two contigs, and then scaffolds are made. Paired-end reads are used again for gap filling of scaffolds to get sequences that have least Ns and cannot be extended on either end. Such sequences are defined as Unigenes. In the final step, blastx alignment (e value <0.00001) between Unigenes and protein databases nr, Swiss-Prot, KEGG and COG is performed, and the best aligning results are used to decide sequence direction of Unigenes. If results of different databases conflict with each other, a priority order of nr, Swiss-Prot, KEGG and COG was followed when deciding sequence direction of Unigenes. When a Unigene happens to be unaligned to none of the above databases, the software ESTScan [37] was introduced to predict its coding regions as well as to decide its sequence direction. Unigene annotation provides information of expression and functional annotation of Unigene. The calculation of Unigene expression uses RPKM method (Reads per kb per million reads) [22]. In the functional annotation, Unigene sequences are firstly aligned by Blastx to protein databases nr, Swiss-Prot, KEGG and COG (E value <0.00001), retrieving proteins with the highest sequence similarity with the given Unigenes along with their protein functional annotations. With nr annotation, we use Blast2GO program [38] to get GO annotation of Unigenes. After getting GO annotation for every Unigene, we use WEGO software [39] to do GO functional classification for all Unigenes and to understand the distribution of gene functions of the species from the macro level.

### mRNA tag (digital gene expression, DGE) library preparation and Illumina sequencing

The main reagents and supplies are Illumina Gene Expression Sample Prep Kit and Illumina Sequencing Chip (flowcell), and the main instruments are Illumina Cluster Station and Illumina HiSeq™ 2000 System. Six micrograms total RNA were extracted, oligo(dT) magnetic beads adsorption was used to purify mRNA, and then Oligo(dT) was used as primer to synthesize the first and second-strand cDNA. The bead-bound cDNA is subsequently digested with restriction enzyme NlaIII, which recognizes and cuts off the CATG sites. The fragments apart from the 3′ cDNA fragments connected to Oligo(dT) beads are washed away and the Illumina adaptor 1 is ligated to the sticky 5′ end of the digested bead-bound cDNA fragments. The junction of Illumina adaptor 1 and CATG site is the recognition site of MmeI, which is a type of endonuclease with separated recognition sites and digestion sites. It cuts at 17 bp downstream of the CATG site, producing tags with adaptor 1. After removing 3′ fragments with magnetic beads precipitation, Illumina adaptor 2 is ligated to the 3′ ends of tags, acquiring tags with different adaptors of both ends to form a tag library. After 15 cycles of linear PCR amplification, 95 bp fragments are purified by 6% TBE PAGE gel electrophoresis. After denaturation, the single-chain molecules are fixed onto the Illumina sequencing chip (flow cell). Each molecule grows into a single-molecule cluster sequencing template through *in situ* amplification. Four types of nucleotides which are labeled by four colors were then added, and sequencing by synthesis (SBS) was performed. Each tunnel will generate millions of raw reads with sequencing length of 35 bp. The DGE datasets are available at the NCBI SRA with the accession number SRX037161.2.

### Analysis and mapping of DGE tags

The pipeline of the DGE bioinformatic analysis is shown in Fig. S6. Sequencing-received raw image data is transformed by base calling into sequence data, which is called raw data or raw reads, and is stored in fastq format. Raw sequences are transformed into clean tags by the following steps: 1. 3′ adaptor sequence removal: since tags are only 21 nt long while the sequencing reads are 35 nt long, raw sequences are with 3′ adaptor sequences; 2. empty reads removal (reads with only 3′ adaptor sequences but no tags); 3. low quality tags removal (tags with unknown sequences ‘N’); 4. removal of tags which are too long or too short, leaving tags of 21 nt long; 5. removal of tags with a copy number of 1 (probable sequencing error). The saturation analysis was performed to check whether the number of detected genes keep increasing when sequencing amount (total tag number) increases. The distribution of clean tag expression was used to evaluate the normality of the whole data. A virtual libraries containing all the possible CATG+17 bases length sequences of the *Taxus* transcriptome sequences was generated. All clean tags were mapped to the reference sequences and only 1 bp mismatch is considered. Clean tags mapped to reference sequences from multiple genes were filtered. Remainder clean tags were designed as unambiguous clean tags. The number of unambiguous clean tags for each gene was calculated and then normalized to TPM (number of transcripts per million clean tags) [17], [32].

### Evaluation of DGE libraries

For screening of differentially expressed genes, a rigorous algorithm to identify differentially expressed genes between two samples was developed based on the previous method [31]. Denote the number of unambiguous clean tag from gene A as x, as every gene's expression occupies only a small part of the library, the p(x) is in the Poisson distribution.


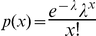
 (λ is the number of the real transcripts of the gene A).

The total clean tag number of the sample 1 is N1, and the total clean tag number of sample 2 is N2; gene A holds x tags in sample 1 and y tags in sample 2. The probability of gene A expressed equally between two samples can be calculated with:


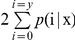
or 
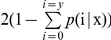
(if
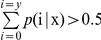
)
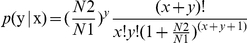



P value corresponds to differential gene expression test. The threshold of p value in multiple tests was determined through manipulating the false discovery rate (FDR) value. Assume that we have picked out R differentially expressed genes (DEGs) in which S genes really show differential expression and the other V genes are false positive. If we decide that the error ratio “Q = V/R” must stay below a cutoff (e.g. 1%), we should preset the FDR to a number no larger than 0.01 [40]. We use “FDR ≤0.001 and the absolute value of log_2_Ratio ≥1” as the threshold to judge the significance of gene expression difference.

The expression annotation of antisense transcripts was performed. Sequencing tags mapped to the complementary strand of the sense gene suggest that its antisense strand also have transcripts, and this gene may use the sense-antisense regulation. In novel transcripts detection, we map the clean tags that can not be mapped to mRNA to the whole genome of *Vitis vinifera* (http://plants.ensembl.org/Vitis_vinifera/Info/Index/), the most closely related species that has whole genome sequence, providing start position that can be uniquely mapped by those tags. We also map these clean tags to the fosmid end sequences of *Taxus mairei* [20], which represent a small portion of *Taxus* genome. We perform cluster analysis of gene expression patterns with Cluster [33] software and Java Treeview [34] software. In gene expression profiling analysis, GO enrichment analysis of functional significance applies hypergeometric test to map all DEGs to terms in GO database, looking for significantly enriched GO terms in DEGs comparing to the *Taxus* transcriptome background. The calculating formula is:
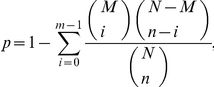



where N is the number of all genes with GO annotation; n is the number of DEGs in N; M is the number of all genes that are annotated to the certain GO terms; m is the number of DEGs in M.

KEGG, the major public pathway-related database, was used in the pathway enrichment analysis to identify significantly enriched metabolic pathways or signal transduction pathways in DEGs comparing with the whole transcriptome background. The calculating formula is the same as that in the GO analysis. Here N is the number of all genes with KEGG annotation, n is the number of DEGs in N, M is the number of all genes annotated to the specific pathways, and m is the number of DEGs in M. The Q value of a test measures the proportion of false positives incurred (i.e., false discovery rate) when that particular test is called significant (http://genomics.princeton.edu/storeylab/qvalue/). Pathways with Q value ≤0.05 are significantly enriched in DEGs.

### Reagents and materials for metabolomics and sample preparation

Millipore water, HPLC grade acetonitrile, and methanol were used throughout; other reagents used were of analytical grade. The authentic standards (purity >98%) including 10-DAB, baccatin III, and paclitaxel were purchased from Sigma. Other taxane standards (purity >96%) used in this study including DAXT, 10-DAT, and cephalomannine were purchased from Shanghai Jinhe Biotechnology Co. Ltd. Plant samples were collected from several yew plantations in China (Tables 5 and 6). All plant samples were collected from trees whose age was known. Yew needles were separated from stem and yew hair roots (i. d. ≤2 mm) and washed with water to remove soil before drying. Yew materials were air-dried (≤50°C), ground to fine particles and sieved through a 40-mesh screen. The extraction and sample preparation were performed as previously described [4], [5].

### Instruments and analytical conditions

All assays were performed on a Shimadzu Prominence UFLC system equipped with a CBM-20A communications bus module, an SIL-20ACHT autosampler, two LC-20AD pumps, a DGU-20A3 vacuum degasser, a CTO-20AC column oven, and an SPD-M 20A diode array detector. A Shim-pack XR-ODS (100 mm×2.0 mm, 2.2 µm; Shimadzu) analytical column with an ODS guard column (5 mm×2.0 mm, 2.2 µm; Shimadzu) was used and kept at 50°C. The mobile phase consisted of water (A) and CH_3_CN (B). The following gradient condition was used: 0–11 min, 69–52.5% A; 11–12 min, 52.5–5% A; 12–15 min, 5% A; and 15–18 min, 69% A. The flow rate was set at 0.4 ml/min, and the injection volume was 1 µl. DAD detection was achieved in the range of 190–370 nm, and the wavelength was set at 230 nm for quantitative analysis. Mass detection and validation of the quantitative analysis were performed as previously described [4], [5].

## Supporting Information

Table S1 **Annotation of 36,493 Unigenes assembled from 13,737,528 Illumina sequence reads.**
(XLS)

Table S2 ***Taxus mairei*** **Unigene sequence alignment with** ***Picea glauca*** **, Korea** ***T. cuspidata*** **, and China** ***T. cuspidate.*** All clone sequences (Closeq) and cluster sequences (Cluseq) are retrieved from http://www.arborea.ulaval.ca/research/sequencing/gene_catalogue/index.html. Clone sequences are all in the direct orientation (5′->3′) and correspond to either ESTs, or assemblies of ESTs when clones produced several ESTs. There is a single clone sequence per clone, which contains its completion status in the FASTA header line. For each cluster, a representative clone is selected (usually the longest and most complete cDNA) to represent a gene. Thus, cluster sequences are nothing else than the clone sequence of their representative clones. QueryX, start position of *Taxus mairei* Unigene sequence that can be aligned with *Picea glauca* sequence (alignment parameters E value ≤1e-5 and the sequence identity ≥80%); QueryY, end position of *T. mairei* Unigene sequence; SbjctX, start position of *P. glauca* sequence that is aligned with *T. mairei* Unigene sequence; SbjctY, end position of *P. glauca* sequence; 5′ read (F), Unigene of *T. mairei* that can be aligned with 5′ CDS sequence of *P. glauca* in the forward orientation; 5′ read (R), Unigene of *T. mairei* that can be aligned with 5′ CDS sequence of *P. glauca* in the reverse orientation; 3′ read (F), Unigene of *T. mairei* that can be aligned with 3′ CDS sequence of *P. glauca* in the forward orientation; 3′ read (R), Unigene of *T. mairei* that can be aligned with 3′ CDS sequence of *P. glauca* in the reverse orientation; is CDS (F), Unigene of *T. mairei* that can be aligned with full length CDS sequence of *P. glauca* in the forward orientation; is CDS (R), Unigene of *T. mairei* that can be aligned with full length CDS sequence of *P. glauca* in the reverse orientation. Unigene of *T. mairei* that can be aligned with the internal (not 5′, 3′, or full length) CDS sequence of *P. glauca* is not annotated with the above six terms.
(XLS)

Table S3 **Genes related to the taxane biosynthetic pathway and metabolism.**
(DOC)

Table S4 **Number of unambiguous clean tags for each gene and normalized TPM (number of transcripts per million clean tags) values in the three DGE libraries.**
(XLS)

Table S5 **Differentially expressed genes between root and leaf.** TPM: transcript copies per million tags. Raw intensity: the total number of tags sequenced for each gene. FDR: false discovery rate. We used FDR ≤0.001 and the absolute value of log_2_Ratio ≥1 as the threshold to judge the significance of gene expression difference. In order to calculate the log_2_Ratio and FDR, we used TPM value of 0.001 instead of 0 for genes that do not express in one sample.
(XLS)

Table S6 **Differentially expressed genes between root and stem.**
(XLS)

Table S7 **Differentially expressed genes between stem and leaf.**
(XLS)

Table S8 **DEGs involved in the taxane biosynthetic pathway.**
(XLS)

Table S9 **Antisense regulation of genes involved in the paclitaxel biosynthetic pathway.**
(DOC)

Table S10 **Novel transcripts detected by mapping DGE tags to the *Taxus* fosmid end sequences (FES) and the *Vitis* genome.**
(DOC)

Table S11 **Clusters containing the taxane biosynthetic genes detected in the clustering analyses of DEGs.**
(XLS)

Table S12 **Pathway enrichment analysis for DEGs (root vs. leaf).**
(DOC)

Table S13 **Quantitative differences in average concentration of six valuable taxanes in the needles of *Taxus mairei* (different age).**
(DOC)

Table S14 **Quantitative differences in average concentration of six valuable taxanes in the roots of *Taxus mairei* (different age).**
(DOC)

Figure S1 **The saturation analysis of the DGE library sequencing.** A, root; B, leaf. The results revealed that with the increase of total sequence number (sequencing depth), the number of new distinct tag decreased markedly.
(TIF)

Figure S2 **Statistics of DGE clean tag alignment.** PM(Sense), perfect match to gene (sense); 1 tag->1 gene, match to one gene; 1 tag->n gene, match to more than one gene; 1 MM(Sense), match to gene (sense) with 1 bp mismatch; PM(AntiSense), perfect match to anti-sense gene; 1 MM(AntiSense), match to anti-sense gene with 1 bp mismatch; PM Genome 1 tag->1 position, perfect match to the *Vitis* genome sequence with one best hit; PM Genome 1 tag->n position, perfect match to the *Vitis* genome sequence with multiple best hits; 1 MM Genome, match to the *Vitis* genome sequence with 1 bp mismatch; Unkown Tag, not match to gene (sense and anti-Sense) and the *Vitis* genome sequence.
(TIF)

Figure S3 **The level of gene expression for each gene.** Gene expression level was determined by calculating the number of unambiguous tags for each gene and then normalizing to TPM (transcript copies per million tags). A, root; B, stem; C, leaf.
(TIF)

Figure S4 **Prediction of protein coding sequence (CDS) from the assembled Unigenes.** Unigenes are firstly aligned by Blastx (E value <0.00001) to protein databases in the priority order of nr, Swiss-Prot, KEGG and COG. Unigenes aligned to databases with higher priority will not enter the next circle. The alignments end when all circles are finished. Proteins with highest ranks in Blast results are taken to decide the coding region sequences of Unigenes, then the coding region sequences are translated into amino acid sequences with the standard codon table. Thus both the nucleotide sequences (5′-3′) and amino acid sequences of the Unigene coding region are acquired. Unigenes that cannot be aligned to any database are scanned by ESTScan (http://www.ch.embnet.org/software/ESTScan.html) to get the nucleotide sequence (5′-3′) and amino acid sequence of the coding regions. A, length distribution of CDS predicted from Blast results and by ESTScan. 1, 200; 2, 300; 3, 400; 4, 500; 5, 600; 6, 700; 7, 800; 8, 900; 9, 1,000; 10, 1,100; 11, 1,200; 12, 1,300; 13, 1,400; 14, 1,500; 15, 1,600; 16, 1,700; 17, 1,800; 18, 1,900; 19, 2,000; 20, 2,100; 21, 2,200; 22, 2,300; 23, 2,400; 24, 2,500; 25, 2,600; 26, 2,700; 27, 2,800; 28, 2,900; 29, 3,000; 30, >3,000. B, gap (N) distribution of CDS predicted from Blast results and by ESTScan. 1, 0; 2, 0.01; 3, 0.02; 4, 0.03; 5, 0.04; 6, 0.05; 7, 0.06; 8, 0.07; 9, 0.08; 10, 0.09; 11, 0.1; 12, 0.11; 13, 0.12; 14, 0.13; 15, 0.14; 16, 0.15; 17, 0.16; 18, 0.17; 19, 0.18; 20, 0.19; 21, 0.2; 22, 0.21; 23, 0.22; 24, 0.23; 25, 0.24; 26, 0.25; 27, 0.26; 28, 0.27; 29, 0.28; 30, 0.29; 31, 0.3; 32, >0.3.
(TIF)

Figure S5 **Pipeline of the transcriptome (mRNA-seq) bioinformatic analysis.**
(TIF)

Figure S6 **Pipeline of the DGE bioinformatic analysis.**
(TIF)
