# Supplementary material for: The First Insight into the Tissue Specific Taxus Transcriptome via Illumina Second Generation Sequencing
Source: PLoS One. 2011 Jun 22;6(6):e21220. doi: 10.1371/journal.pone.0021220 (PMC3120849; doi:10.1371/journal.pone.0021220)
Supplement: Table S3 — Genes related to the taxane biosynthetic pathway and metabolism. (DOC) [file pone.0021220.s003.doc]

Table S3 Genes related to the taxane biosynthetic pathway and metabolism

|  | Unigene (RPKM) | Abundance (sum of RPKM) | No. of sequences |
| --- | --- | --- | --- |
| Geranylgeranyl diphosphate synthase (GGPPS) | 35278(152.7676) 30791(254.4094) 15203(20.5345) 32201(80.7439) 28798(11.8242) 1460(8.2122) 21802(80.4858) 5427(12.7707) 34156(22.3702) 33137(140.0361) 579(3.602) | 787.7566 | 11 |
| Taxadiene synthase (TS) | 24106(671.7171) 27160(203.7264) 27766(690.1409) 28505(174.2462) 28907(229.9197) 33259(136.8129) | 2106.5632 | 6 |
| Taxadiene 5α hydroxylase (T5OH) | 11246(8.6313) 25527(52.8105) 4497(21.0697) 4712(33.7077) 11020(29.4436) 25555(121.7668) 33175(58.1918) 28660(16.5425) 891(45.6252) | 387.7891 | 9 |
| Taxadien-5α-ol O-acetyltransferase (TAT) | 10440(7.7678) 19196(20.2779) 19606(35.3377) 13264(37.351) 29438(138.465) 32910(102.7165) | 341.9159 | 6 |
| Taxane 10β hydroxylase (T10OH) | 1152(57.134) 13330(49.0385) 1373(38.5376) 15644(25.3092) 34599(68.9537) 4908(23.1024) 9194(54.9919) 16140(8.9692) 17170(3.4579) 25561(38.6828) 5260(13.8502) 6686(14.7664) 8006(36.399) 8115(21.8494) 13158(14.0918) 1189(18.6835) 11897(13.4317) 1430(24.5846) 14790(28.7603) 1481(9.8236) 14871(38.0216) 14905(31.2615) 15110(16.3705) 15620(5.5053) 15651(58.8641) 15955(50.3153) 16040(30.5336) 17407(51.1972) 1636(13.7051) 18050(33.1982) 20099(28.6415) 20311(19.3944) 2060(130.2105) 21734(67.6175) 2173(54.9998) 21837(51.5279) 23425(117.5689) 23761(3.775) 24203(65.9347) 24959(99.7474) 25250(224.3524) 25264(44.9391) 25369(125.9278) 26700(77.3972) 28608(97.6886) 2891(6.9074) 29765(97.0135) 31188(141.7009) 32608(126.0765) 32767(71.9584) 3601(53.4307) 7101(11.0859) | 2611.2861 | 52 |
| Taxane 13α hydroxylase (T13OH) | 6456(20.0232) 14074(17.199) 21821(17.8857) 24247(13.8608) 26412(54.2292) 30266(51.6411) 33001(101.9801) | 276.8191 | 7 |
| Taxoid 2α hydroxylase (T2OH) | 14478(44.0953) 22960(184.7919) 25426(73.9043) 35029(54.6935) | 357.485 | 4 |
| Taxoid 7β hydroxylase (T7OH) | 17688(38.4266) | 38.4266 | 1 |
| 2α-hydroxytaxane 2-O-benzoyltransferase (TBT) | 10346(9.0377) 10994(13.6809) 13698(7.204) 22626(282.0712) 33891(53.4453) 4988(8.1132) 2582(17.3572) 31915(49.08) 5485(5.2552) 856(27.7868) | 473.0315 | 10 |
| 10-Deacetylbaccatin III 10-O-acetyltransferase (DBAT) | 11135(16.4418) 1879(11.7829) 28657(24.2801) 25786(7.9492) | 60.454 | 4 |
| Phenylpropanoyl transferase (BAPT) | 11534(12.5021) 23514(8.0328) 25575(94.5207) 31755(119.8135) | 234.8691 | 4 |
| 3’-N-debenzoyl-2’-deoxytaxol N-benzoyltransferase (DBTNBT) | 11254(7.46) 15359(25.1188) 15942(25.9343) 18136(60.7864) 22418(28.8159) 24045(47.2876) 24051(44.3322) 28058(65.8917) 4407(3.4531) 4793(16.4091) 4794(39.2216) 4838(6.5096) 5777(55.1127) 8752(8.1717) | 434.5047 | 14 |
| Phenylalanine aminomutase (PAM) | 21200(44.845) | 44.845 | 1 |
| Taxane 14β hydroxylase (T14OH) | 24981(133.8545) 29010(193.5397) 29803(222.7046) 4576(52.0545) 4847(13.8666) 487(7.2357) | 623.2556 | 6 |
| Xylosyltransferase (XYLT) | 12767 (30.9211) 25564 (174.2409) 32554 (215.4439) 32672 (165.2774) 35019 (23.8961) 36198 (78.6198) | 688.3992 | 6 |
| Upstream methylerythritol-phosphate (MEP) pathway |  |  |  |
| 1-Deoxy-D-xylulose-5-phosphate synthase (DXS) | 23718(148.7354) 23813(100.7304) 24752(155.8893) 26668(137.502) 34970(180.9775) 35249(73.4572) 36307(74.3022) 5532(39.0659) | 910.6599 | 8 |
| 1-Deoxy-D-xylulose 5-phosphate reductoisomerase (DXR) | 21220(50.5371) 21533(61.3779) 34499(56.3891) | 168.3041 | 3 |
| 2-C-methyl-D-erythritol 4-phosphate cytidyltransferase (CMS) | 11120(33.5582) | 33.5582 | 1 |
| 4-Diphosphocytidyl-2-C-methyl-D-erythritol kinase (CMK) | 16063(45.6705) | 45.6705 | 1 |
| 2-C-methyl-D-erythritol 2,4-cyclodiphosphate synthase (MCS) | 11816(19.53) 22134(116.0989) | 135.6289 | 2 |
| 4-Hydroxy-3-methylbut-2-en-1-yl diphosphate synthase (HDS) | 30749(151.4996) 32120(127.6905) 33401(128.8671) 24492(88.2487) | 496.3059 | 4 |
| 4-Hydroxy-3-methylbut-2-enyl diphosphate reductase (HDR) | 34021(185.0033) 35163(180.7718) | 365.7751 | 2 |
| Isopentenyl-diphosphate isomerase (IDI) | 27971(63.7729) 34622(109.5004) | 173.2733 | 2 |

The calculation of Unigene expression uses RPKM method (Reads Per kb per Million reads) [18], the formula is shown below:

$$RPKM=\frac{10^{6}C}{NL/10^{3}}$$

Set RPKM(A) to be the expression of Unigene A, and C to be number of reads that uniquely aligned to Unigene A, N to be total number of reads that uniquely aligned to all Unigenes, and L to be number of bases on Unigene A. The RPKM method is able to eliminate the influence of different gene length and sequencing level on the calculation of gene expression. Therefore the calculated gene expression can be directly used for comparing the difference of gene expression.
