# Supplementary material for: The First Insight into the Tissue Specific Taxus Transcriptome via Illumina Second Generation Sequencing
Source: PLoS One. 2011 Jun 22;6(6):e21220. doi: 10.1371/journal.pone.0021220 (PMC3120849; doi:10.1371/journal.pone.0021220)
Supplement: Table S9 — Antisense regulation of genes involved in the paclitaxel biosynthetic pathway. (DOC) [file pone.0021220.s009.doc]

Table S9 Antisense regulation of genes involved in the taxane biosynthetic pathway

|  | | Root | Stem | Leaf |
| --- | --- | --- | --- | --- |
| TPM(+) | Max | 185.19 | 346.79 | 275.2 |
|  | Min | 0 | 0 | 0 |
|  | Median | 8.56 | 6.26 | 5.5 |
| TPM(-) | Max | 10.15 | 4.47 | 6.95 |
|  | Min | 0.63 | 0.6 | 0.58 |
|  | Median | 1.59 | 1.19 | 1.16 |
| TPM(+)  /TPM(-) | >1 | 33 | 17 | 23 |
|  | ≤1 | 9 | 8 | 14 |
| No. of Unigenes | | 42 | 25 | 37 |
