# Supplementary material for: The First Insight into the Tissue Specific Taxus Transcriptome via Illumina Second Generation Sequencing
Source: PLoS One. 2011 Jun 22;6(6):e21220. doi: 10.1371/journal.pone.0021220 (PMC3120849; doi:10.1371/journal.pone.0021220)
Supplement: Table S10 — Novel transcripts detected by mapping DGE tags to the Taxus fosmid end sequences (FES) and the Vitis genome. (DOC) [file pone.0021220.s010.doc]

Table S10 Novel transcripts detected by mapping DGE tags to the *Taxus* fosmid end sequences (FES) and the *Vitis* genome

|  | | Root | Stem | Leaf |
| --- | --- | --- | --- | --- |
| No. of novel transcripts | FES mapping | 1106 | 702 | 748 |
|  | Vitis mapping | 8614 | 5220 | 5242 |
| One mismatch | FES mapping | 794 | 482 | 508 |
|  | Vitis mapping | 8483 | 5138 | 5166 |
| No mismatch | FES mapping | 312 | 220 | 240 |
|  | Vitis mapping | 131 | 82 | 76 |
| Unique hit | FES mapping | 848 | 546 | 563 |
|  | Vitis mapping | 5654 | 3396 | 3520 |
| Two hits | FES mapping | 258 | 156 | 185 |
|  | Vitis mapping | 2960 | 1824 | 1722 |
| + | FES mapping | 552 | 354 | 368 |
|  | Vitis mapping | 4301 | 2647 | 2641 |
| - | FES mapping | 554 | 348 | 380 |
|  | Vitis mapping | 4313 | 2573 | 2601 |
| TPM>5.623 | FES mapping | 42 | 36 | 51 |
|  | Vitis mapping | 1027 | 742 | 693 |
| TPM<5.623 | FES mapping | 1064 | 666 | 697 |
|  | Vitis mapping | 7587 | 4478 | 4549 |

TPM, normalized expression level of tags; hits, number of locations on FES/genome aligned by tags; +/-, alignment on the direct (+) or reverse (-) chain of the reference; 5.623, average TPM value of novel transcripts detected by mapping DGE tags to the *Vitis* genome.
