# Supplementary material for: The First Insight into the Tissue Specific Taxus Transcriptome via Illumina Second Generation Sequencing
Source: PLoS One. 2011 Jun 22;6(6):e21220. doi: 10.1371/journal.pone.0021220 (PMC3120849; doi:10.1371/journal.pone.0021220)
Supplement: Table S12 — Pathway enrichment analysis for DEGs (root vs. leaf). (DOC) [file pone.0021220.s012.doc]

Table S12 Pathway enrichment analysis for DEGs (root vs. leaf)

| **#** | Pathway | DEGs with pathway annotation (1901) | All genes with pathway annotation (11550) | P value | Q value | Pathway ID |
| --- | --- | --- | --- | --- | --- | --- |
| 1 | Ribosome | 81 (4.26%) | 264 (2.29%) | 4.999967e-09 | 5.749962e-07 | ko03010 |
| 2 | Biosynthesis of  secondary metabolites | 292 (15.36%) | 1,404 (12.16%) | 3.07746e-06 | 1.189681e-04 | ko01110 |
| 3 | Photosynthesis | 25 (1.32%) | 60 (0.52%) | 3.103516e-06 | 1.189681e-04 | ko00195 |
| 4 | Phenylpropanoid biosynthesis | 71 (3.73%) | 274 (2.37%) | 3.788265e-05 | 8.794970e-04 | ko00940 |
| 5 | Metabolic pathways | 503 (26.46%) | 2,647 (22.92%) | 4.066554e-05 | 8.794970e-04 | ko01100 |
| 6 | Phenylalanine metabolism | 39 (2.05%) | 127 (1.1%) | 4.58868e-05 | 8.794970e-04 | ko00360 |
| 7 | Fructose and  mannose metabolism | 27 (1.42%) | 81 (0.7%) | 0.0001456205 | 2.392337e-03 | ko00051 |
| 8 | Amino sugar and nucleotide  sugar metabolism | 40 (2.1%) | 149 (1.29%) | 0.0008455015 | 1.215408e-02 | ko00520 |
| 9 | Glutathione metabolism | 28 (1.47%) | 95 (0.82%) | 0.001059832 | 1.354230e-02 | ko00480 |
| 10 | Pentose and glucuronate interconversions | 28 (1.47%) | 97 (0.84%) | 0.001505170 | 1.730945e-02 | ko00040 |
| 11 | Flavonoid biosynthesis | 41 (2.16%) | 162 (1.4%) | 0.002486857 | 2.599896e-02 | ko00941 |
| 12 | Ascorbate and  aldarate metabolism | 23 (1.21%) | 81 (0.7%) | 0.004772038 | 4.573203e-02 | ko00053 |
| 13 | Glyoxylate and  dicarboxylate metabolism | 15 (0.79%) | 46 (0.4%) | 0.005290394 | 4.679964e-02 | ko00630 |

Pathways with Q value ≤0.05 are significantly enriched in DEGs.
