# Supplementary material for: The First Insight into the Tissue Specific Taxus Transcriptome via Illumina Second Generation Sequencing
Source: PLoS One. 2011 Jun 22;6(6):e21220. doi: 10.1371/journal.pone.0021220 (PMC3120849; doi:10.1371/journal.pone.0021220)
Supplement: Table S13 — Quantitative differences in average concentration of six valuable taxanes in the needles of Taxus mairei (different age). (DOC) [file pone.0021220.s013.doc]

Table S12 Quantitative differences in average concentration of six valuable taxanes in the needles of *Taxus mairei* (different age)

| Compound | Age of *Taxus* | | | | | | | |
| --- | --- | --- | --- | --- | --- | --- | --- | --- |
|  | 3 yrs | 4 yrs | 5 yrs | 6 yrs | 7 yrs | average | SD | RSD (%) |
| DAB | 168.2 | 104.6 | 87.9 | 70.5 | 85.4 | 103.3 | 34.2 | 33.1 |
| B | 25.9 | 21.6 | 14.0 | 10.6 | 9.9 | 16.4 | 6.3 | 38.3 |
| DAXT | 341.3 | 576.7 | 558.4 | 513.1 | 539.5 | 505.8 | 84.9 | 16.8 |
| DAT | 157.7 | 170.3 | 158.7 | 66.2 | 125.9 | 135.7 | 37.8 | 27.8 |
| C | 73.0 | 84.5 | 107.2 | 35.7 | 106.8 | 81.4 | 26.4 | 32.4 |
| P | 52.7 | 82.1 | 84.5 | 22.3 | 53.7 | 59.1 | 22.8 | 38.6 |
| Total (μg/g dry needle) | 818.8 | 1039.8 | 1010.7 | 718.4 | 921.2 | 901.7 |  |  |

SD, standard deviation; RSD, relative standard deviation. RSD = 100SD/average.

Three samples were collected from three individual yews at each age.

DAB, 10-deacetylbaccatin III; B, baccatin III; DAXT, 7-xylosyl-10-deacetylpaclitaxel; DAT, 10-deacetylpaclitaxel; C, cephalomannine; P, paclitaxel.
