# Supplementary material for: The First Insight into the Tissue Specific Taxus Transcriptome via Illumina Second Generation Sequencing
Source: PLoS One. 2011 Jun 22;6(6):e21220. doi: 10.1371/journal.pone.0021220 (PMC3120849; doi:10.1371/journal.pone.0021220)
Supplement: Table S14 — Quantitative differences in average concentration of six valuable taxanes in the roots of Taxus mairei (different age). (DOC) [file pone.0021220.s014.doc]

Table S13 Quantitative differences in average concentration of six valuable taxanes in the roots of *Taxus mairei* (different age)

| Compound | Age of *Taxus* | | | | | | | |
| --- | --- | --- | --- | --- | --- | --- | --- | --- |
|  | 3 yrs | 4 yrs | 5 yrs | 6 yrs | 7 yrs | average | SD | RSD (%) |
| DAB | 125.0 | 177.6 | 56.4 | 87.4 | 195.6 | 128.4 | 52.6 | 40.9 |
| B | 200.6 | 290.2 | 165.8 | 248.2 | 150.5 | 211.1 | 51.9 | 24.6 |
| DAXT | 243.0 | 544.6 | 209.5 | 519.0 | 802.5 | 463.7 | 218.0 | 47.0 |
| DAT | 226.6 | 681.4 | 121.4 | 361.6 | 280.3 | 334.3 | 190.3 | 56.9 |
| C | 231.3 | 361.6 | 155.9 | 182.0 | 246.1 | 235.4 | 71.0 | 30.2 |
| P | 329.9 | 480.6 | 103.3 | 360.0 | 406.9 | 336.1 | 127.1 | 37.8 |
| Total (μg/g dry powder) | 1356.5 | 2056.0 | 709.3 | 1398.2 | 1675.1 | 1439.0 |  |  |

SD, standard deviation; RSD, relative standard deviation. RSD = 100SD/average.

Three samples were collected from three individual yews at each age.
