# Supplementary figures and images for: The First Insight into the Tissue Specific Taxus Transcriptome via Illumina Second Generation Sequencing
Source: PLoS One. 2011 Jun 22;6(6):e21220. doi: 10.1371/journal.pone.0021220 (PMC3120849; doi:10.1371/journal.pone.0021220)

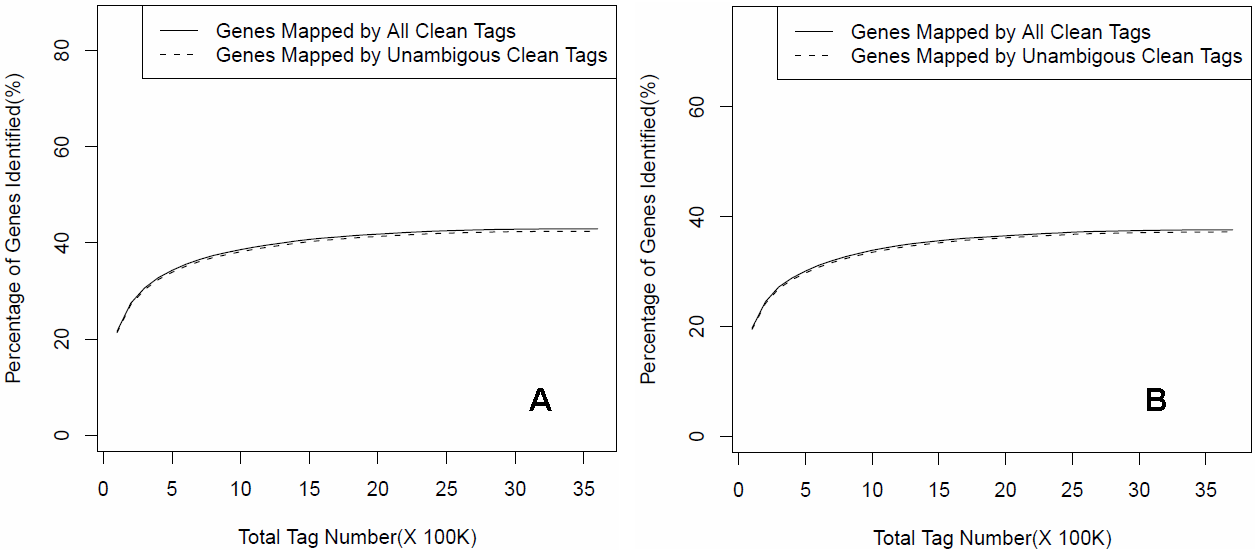

Supplement: Figure S1 — The saturation analysis of the DGE library sequencing. A, root; B, leaf. The results revealed that with the increase of total sequence number (sequencing depth), the number of new distinct tag decreased markedly. (TIF) [file pone.0021220.s015.tif]

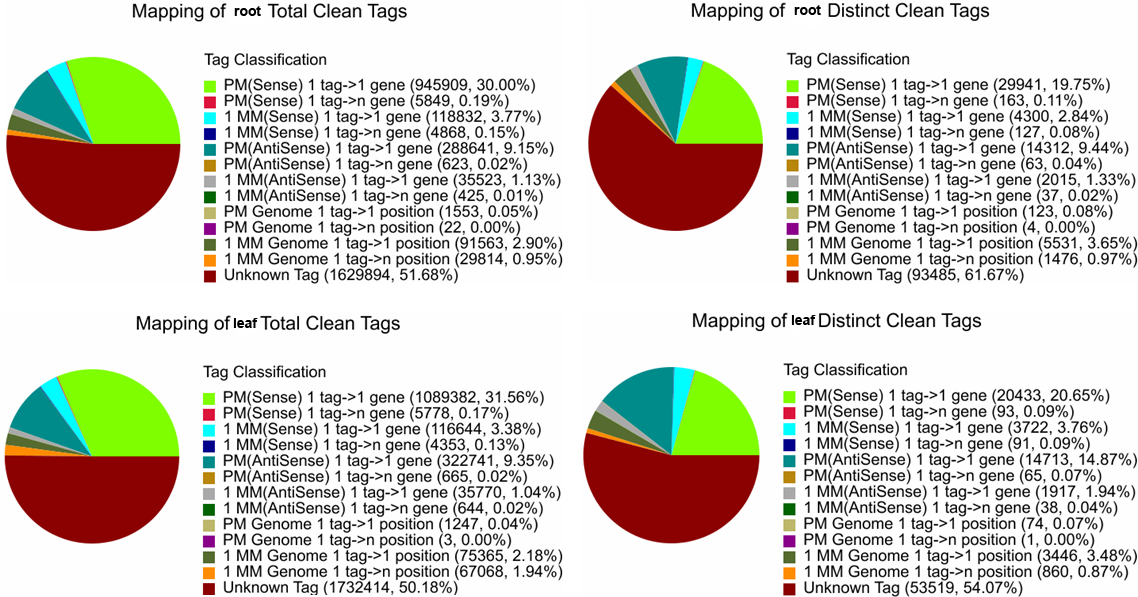

Supplement: Figure S2 — Statistics of DGE clean tag alignment. PM(Sense), perfect match to gene (sense); 1 tag->1 gene, match to one gene; 1 tag->n gene, match to more than one gene; 1 MM(Sense), match to gene (sense) with 1 bp mismatch; PM(AntiSense), perfect match to anti-sense gene; 1 MM(AntiSense), match to anti-sense gene with 1 bp mismatch; PM Genome 1 tag->1 position, perfect match to the Vitis genome sequence with one best hit; PM Genome 1 tag->n position, perfect match to the Vitis genome sequence with multiple best hits; 1 MM Genome, match to the Vitis genome sequence with 1 bp mismatch; Unkown Tag, not match to gene (sense and anti-Sense) and the Vitis genome sequence. (TIF) [file pone.0021220.s016.tif]

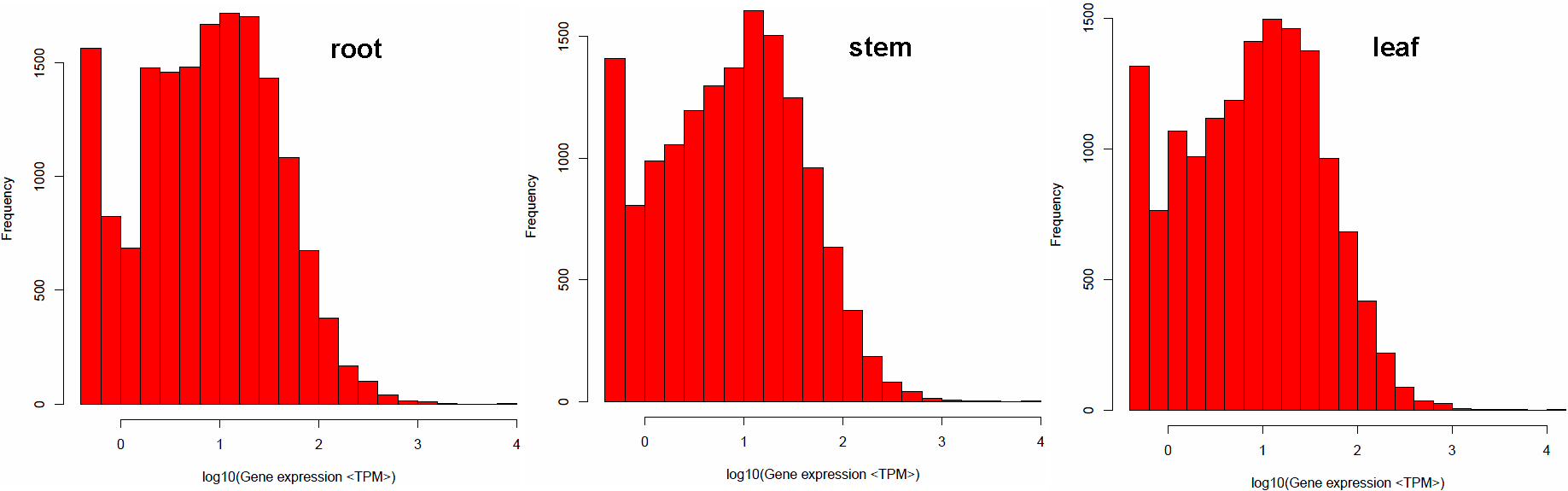

Supplement: Figure S3 — The level of gene expression for each gene. Gene expression level was determined by calculating the number of unambiguous tags for each gene and then normalizing to TPM (transcript copies per million tags). A, root; B, stem; C, leaf. (TIF) [file pone.0021220.s017.tif]

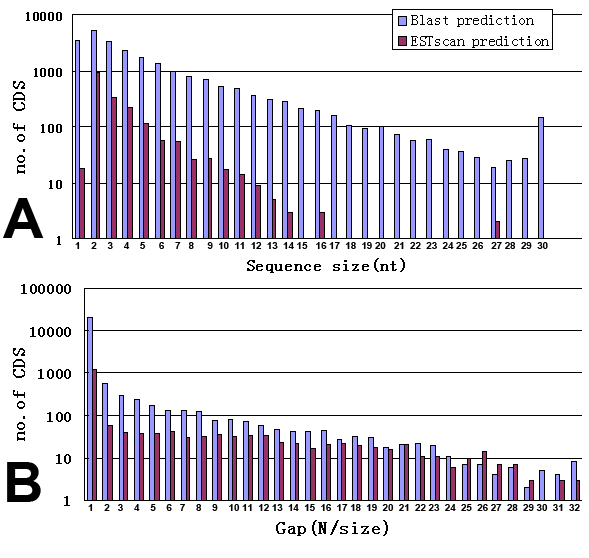

Supplement: Figure S4 — Prediction of protein coding sequence (CDS) from the assembled Unigenes. Unigenes are firstly aligned by Blastx (E value <0.00001) to protein databases in the priority order of nr, Swiss-Prot, KEGG and COG. Unigenes aligned to databases with higher priority will not enter the next circle. The alignments end when all circles are finished. Proteins with highest ranks in Blast results are taken to decide the coding region sequences of Unigenes, then the coding region sequences are translated into amino acid sequences with the standard codon table. Thus both the nucleotide sequences (5′-3′) and amino acid sequences of the Unigene coding region are acquired. Unigenes that cannot be aligned to any database are scanned by ESTScan (http://www.ch.embnet.org/software/ESTScan.html) to get the nucleotide sequence (5′-3′) and amino acid sequence of the coding regions. A, length distribution of CDS predicted from Blast results and by ESTScan. 1, 200; 2, 300; 3, 400; 4, 500; 5, 600; 6, 700; 7, 800; 8, 900; 9, 1,000; 10, 1,100; 11, 1,200; 12, 1,300; 13, 1,400; 14, 1,500; 15, 1,600; 16, 1,700; 17, 1,800; 18, 1,900; 19, 2,000; 20, 2,100; 21, 2,200; 22, 2,300; 23, 2,400; 24, 2,500; 25, 2,600; 26, 2,700; 27, 2,800; 28, 2,900; 29, 3,000; 30, >3,000. B, gap (N) distribution of CDS predicted from Blast results and by ESTScan. 1, 0; 2, 0.01; 3, 0.02; 4, 0.03; 5, 0.04; 6, 0.05; 7, 0.06; 8, 0.07; 9, 0.08; 10, 0.09; 11, 0.1; 12, 0.11; 13, 0.12; 14, 0.13; 15, 0.14; 16, 0.15; 17, 0.16; 18, 0.17; 19, 0.18; 20, 0.19; 21, 0.2; 22, 0.21; 23, 0.22; 24, 0.23; 25, 0.24; 26, 0.25; 27, 0.26; 28, 0.27; 29, 0.28; 30, 0.29; 31, 0.3; 32, >0.3. (TIF) [file pone.0021220.s018.tif]

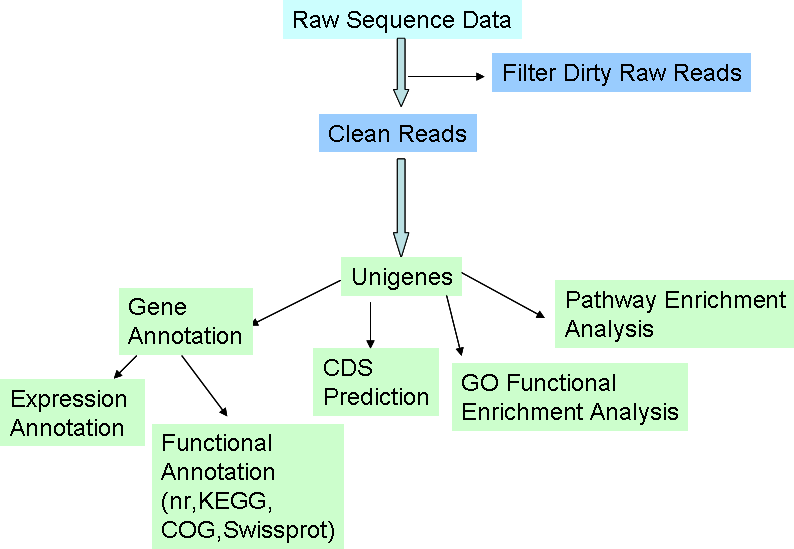

Supplement: Figure S5 — Pipeline of the transcriptome (mRNA-seq) bioinformatic analysis. (TIF) [file pone.0021220.s019.tif]

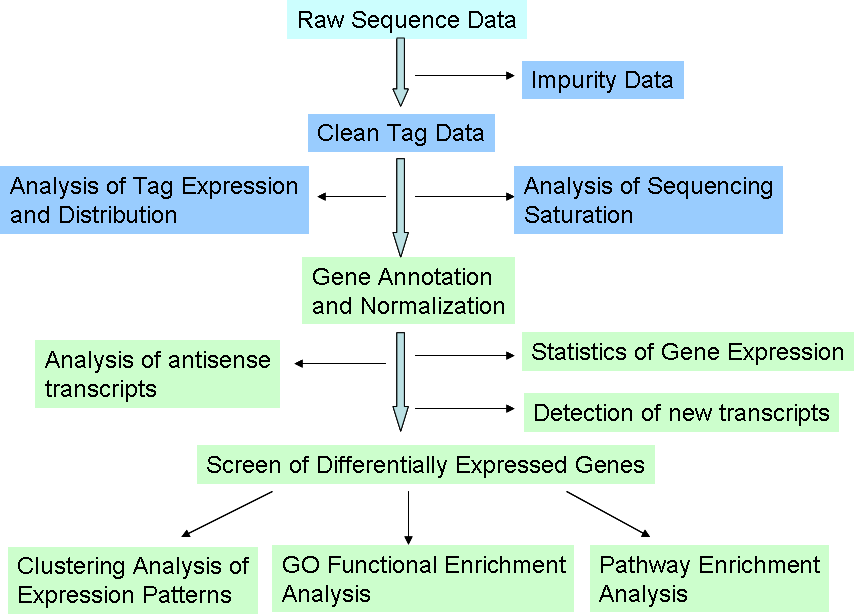

Supplement: Figure S6 — Pipeline of the DGE bioinformatic analysis. (TIF) [file pone.0021220.s020.tif]
